# Taste coding of heavy metal ion-induced avoidance in *Drosophila*

**DOI:** 10.1016/j.isci.2023.106607

**Published:** 2023-04-07

**Authors:** Xiaonan Li, Yuanjie Sun, Shan Gao, Yan Li, Li Liu, Yan Zhu

**Affiliations:** 1 State Key Laboratory of Brain and Cognitive Science, Institute of Biophysics, Chinese Academy of Sciences, 15 Datun Road, Beijing 100101, China; 2 University of Chinese Academy of Sciences, Beijing 100049, China; 3 Advanced Innovation Center for Human Brain Protection, Capital Medical University, Beijing, China

**Keywords:** Environmental toxicology, Neuroscience

## Abstract

Increasing pollution of heavy metals poses great risks to animals globally. Their survival likely relies on an ability to detect and avoid harmful heavy metal ions (HMIs). Currently, little is known about the neural mechanisms of HMI detection. Here, we show that *Drosophila* and related species of *Drosophilidae* actively avoid toxic HMIs at micromolar concentrations. The high sensitivity to HMIs is biologically relevant. Particularly, their sensitivity to cadmium is as high as that to the most bitter substance, denatonium. Detection of HMIs in food requires *Gr66a*^*+*^ gustatory neurons but is independent of bitter-taste receptors. In these neurons, the ionotropic receptors IR76b, IR25a, and IR7a are required for the perception of heavy metals. Furthermore, IR47a mediates the activation of a distinct group of non-*Gr66a*^*+*^ gustatory neurons elicited by HMIs. Together, our findings reveal a surprising taste quality represented by noxious metal ions.

## Highlights

• *Drosophilidae* family has unusual capabilities to avoid detrimental heavy metal ions
• Aversive sensitivity to Cd^2+^ is comparable to that of the most bitter compound
• Metallic sensation involving parallel pathways is distinct from bitterant sensation
• Toxic heavy metal ions activate gustatory neurons via IR76b, IR25a, IR7a, and IR47a

## Graphical abstract

**Figure:**
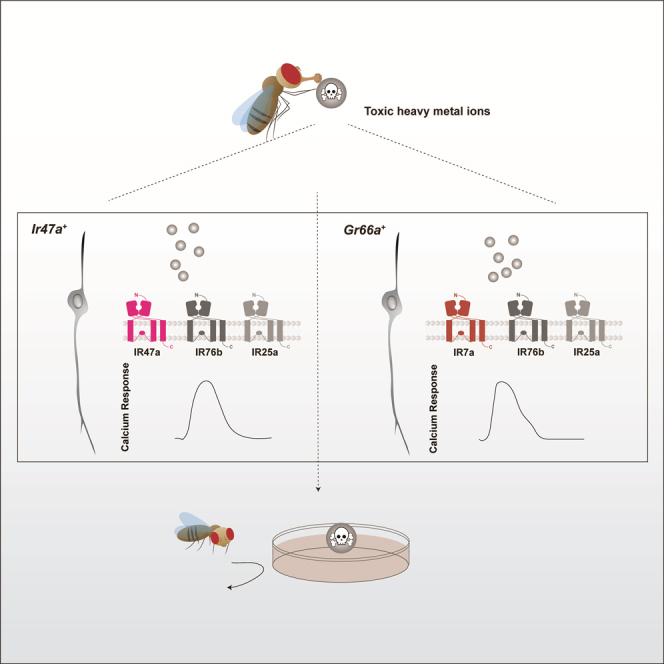

## Introduction

Heavy metals are commonly defined as metal elements with a relatively high molecular weight and density. About 70% of the elements in the periodic table are heavy metals. Weathering and volcanic eruptions contribute significantly to the natural occurrence of heavy metal pollution. However, most environmental contamination and human exposure to heavy metals are the results of anthropogenic activities such as mining, industrial production, and the use of metals and related compounds. Heavy metal pollution has severely threatened humans, animals, and plants.^1^

Some heavy metals, such as iron (Fe), copper (Cu), cobalt (Co), and zinc (Zn), are essential nutrients and are required in physiological processes,^2^ whereas other metals, such as cadmium (Cd), mercury (Hg), and lead (Pb), are highly toxic or carcinogenic to organisms.^3^ For most individuals, diet is the largest source of exposure to heavy metals. Although related mechanisms are still not well-understood, bioaccumulation of heavy metals is known to interfere with normal functions, induce cancer, and damage organs, including the heart, intestines, kidneys, reproductive system, and nervous system.^3^^,^^4^^,^^5^ Toxic heavy metals are also associated with the progression of neurodegenerative diseases, including Alzheimer’s disease.^6^

Assessing ingredients in food via sensory systems is critical to avoid ingesting toxins or harmful substances.^7^ Many species, ranging from worms^8^ to mammals,^9^ develop taste sensations for heavy metal ions (HMIs). Humans have a complex taste profile of HMIs. Divalent and trivalent HMIs, such as Fe^2+^, Zn^2+^, and Cu^2+^, are commonly known to elicit bitter, salty, and astringent tastes.^10^^,^^11^ The metallic taste of Fe^2+^ is due to a retronasal smell.^10^^,^^12^ While mercury salts have a metallic taste, lead acetate has a sweet taste.^13^ A recent study suggested that a G protein-coupled receptor (GPCR) in humans, taste 2 receptor member 7 (TAS2R7), mediates the bitterness of multiple HMIs *in vitro.*^14^ In mice, calcium (Ca^2+^) and magnesium (Mg^2+^) activate T1R3, a GPCR expressed in fungiform taste buds.^15^^,^^16^ FeSO_4_ or ZnSO_4_ activate taste system through the T1R3-TRPM5 pathway at low concentrations, and through a member of the transient receptor potential (TRP) family, TRPV1, at high concentrations.^17^ However, the ability and mechanism of sensing other HMIs, especially those with higher molecular weight, are little investigated in mammals, as well as in other organisms.

*Drosophila* has multiple taste modalities and associated behavioral responses.^18^^,^^19^^,^^20^ It was demonstrated that adult flies, as well as larvae, tended to stay away from food containing a high concentration of several HMIs, although responsible receptors and neurons have not been reported.^21^ In recent years, ionotropic receptors (IRs),^22^ were reported to also mediate taste sensation, including the detection of amino acid and fatty acid.^23^^,^^24^^,^^25^ Flies were shown to detect sodium (Na^+^), Ca^2+^ and several essential elements via IRs. Low Na^+^ is attractive, whereas high Na^+^ is repulsive.^26^ Loss of *Ir76b* reverted attraction to repulsion at low Na^+^. Additionally, a rejective response to Ca^2+^ is mediated by IR25a, IR62a, and IR76b.^27^ Very recently, the rejective response to Zn^2+^, an essential element, was shown to be mediated by IR25a, IR76b, and IR56b expressed in *ppk23*^*+*^ neurons.^28^ However, gustatory avoidance of different essential HMIs seems to rely on different receptors because GR66a and GR33a were required for the aversive taste response to Cu^2+^, while IR76b, IR25a, and IR56b were not.^29^ Toxic HMIs possess properties very distinct from those of Na^+^ or Ca^2+^, which are abundant, easily accessible, and essential for normal cellular functions. Indeed, trace amounts of essential elements such as Zn^2+^ or Cu^2+^ are indispensable for the well-being of organisms, but venomous after excessive intake. It is not clear whether a similar IR-mediated mechanism applies to toxic HMIs.

Studies on metallic sensation in mammals and flies have generally utilized HMIs at concentrations in the millimolar range. However, a contaminated area could also give rise to far larger areas with lower but still potentially harmful concentrations; thus animals with higher sensitivity should have a higher chance of detection and avoidance, resulting in adaptive advantages. To address the questions above, we investigated the behavioral, cellular, and molecular basis for heavy metal taste in *Drosophila*. We found that flies strongly avoid toxic HMIs at the micromolar level, which is comparable to their sensitivity to common bitter agents. This high sensitivity helps to protect flies from HMIs-contaminated food. Both *Gr66a*^+^ neurons and non-*Gr66a*^*+*^ neurons in the labellum are activated by Cd^2+^. Furthermore, IRs, instead of GRs, in these neurons are required for the proper detection of HMIs. Our results demonstrate that *Drosophila* is able to sense a broad spectrum of HMIs with high sensitivity.

## Results

### Robust aversive response to heavy metal ions

**Figure 1:**
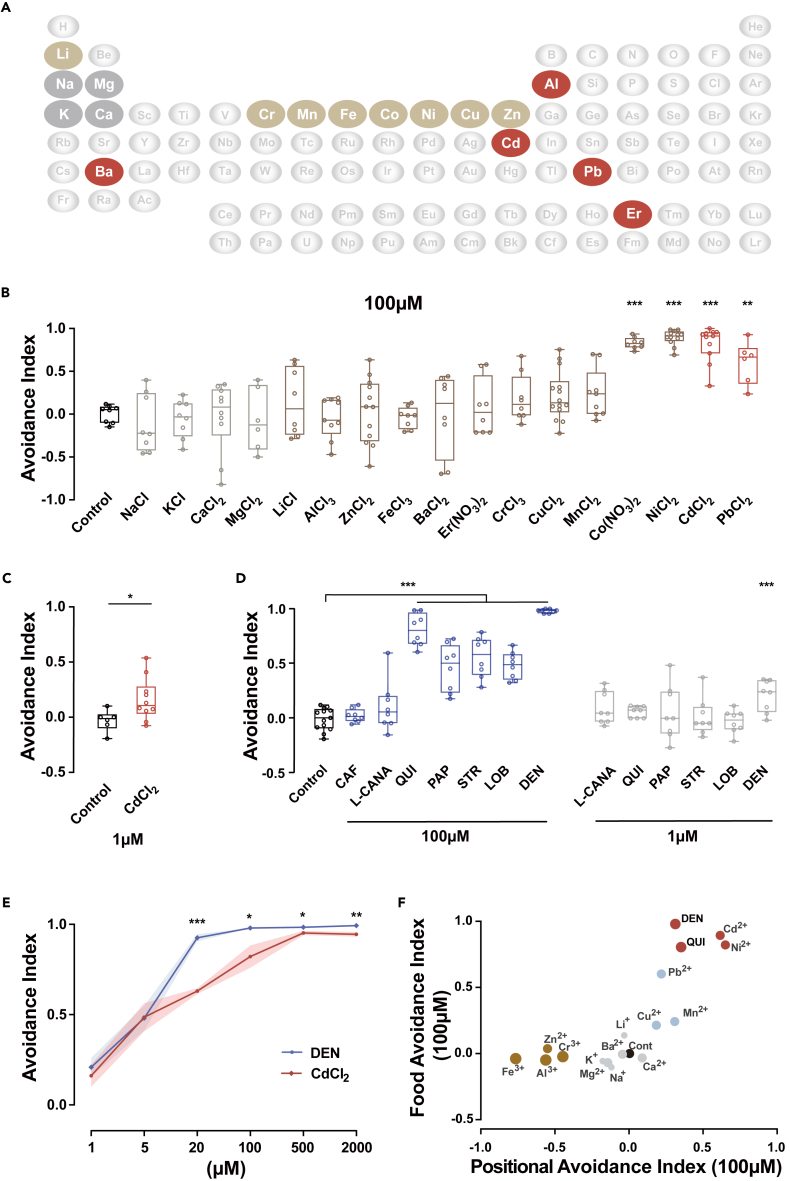
Wild-type flies avoid medium containing heavy metals in a two-choice assay (A) Periodic table of the elements highlighting the metal elements to be tested. Gray: essential metal, brown: trace metal, red: toxic HMIs. (B) Quantification of avoidance of *Canton-S* flies to food containing metal ions (100 μM) in a feeding-choice assay. Feeding preferences of flies was based on the food colors in abdomens and dyes were switched for each metal ion tested to eliminate the preference for dyes. N = 8–12. (C) Quantification of *Canton-S* avoidance of Cd^2+^ at 1 μM. N = 6–10. (D) Quantification of *Canton-S* avoidance of seven bitterants at 100 μM and 1 μM. Full names of bitterants are included in the abbreviation list. N = 8. (E) Comparing the strength of repulsion between Cd^2+^ and DEN at different concentrations. Data are represented as mean ± SEM. N = 8. (F) Cluster analysis (K-means, K = 4) grouped metal ions and bitterants according to the evoked avoidance responses in feeding-choice and positional-choice assays. Four clusters were labeled by gray, brown, blue, and red. The control group was presented with a black spot in the origin. The charges of metal ions are indicated by the sizes of spots. Box and whisker plots in B–D: the scatter points show all data points; the box includes the 25th to 75th percentile, and the line in the box shows the median of the dataset. Statistical analyses compared stimulus and control groups. No metal ions were added to the food in the control groups. One-way ANOVA followed by Tukey’s post hoc test for multiple comparisons in (B) and (D). *∗*p < 0.05, *∗∗*p < 0.01, *∗∗∗*p < 0.001. See also Figures S1 and S2.

Metals broadly exist in the natural environment. In many locations, HMIs contaminate soil, water, and food with concentrations sufficient to cause physiological and mental damage. Thus, the ability to detect toxic ions before ingestion would be an admissible survival advantage for an animal. To systematically investigate the perception of HMIs by the common fruit fly, a food-choice assay was used to quantify their preference for food with various HMIs (Figures S1A and S1B). From the periodic table of elements, 17 representative elements were selected for a basic survey (Figure 1A). These elements represent four classes: essential elements for humans or animals, including Na, potassium (K), Ca, and Mg; essential trace elements, including lithium (Li), chromium (Cr), manganese (Mn), nickel (Ni), Fe, Co, Cu, and Zn; toxic metals, including aluminum (Al), barium (Ba), Cd, and Pb; and a lanthanide rare-earth element, erbium (Er) (Figure 1A). Among these, Cr, Mn, Fe, Co, Ni, Cu, Zn, Cd, Ba, Pb, and Er are heavy metal elements.^30^

At 0.1 mM, wild-type flies showed no bias toward the ions of four essential elements and most of the trace elements. However, ions of trace elements (Co^2+^) induced significant avoidance behavior (Figure 1B). Interestingly, flies strongly dislike food containing Ni^2+^, Cd^2+^, and Pb^2+^ (Figure 1B). The contributions from the common anionic groups in these compounds, Cl^−^ or NO_3_^−^, are negligible. These results suggest that flies can detect and discriminate HMIs in food.

Previous reports evaluated responses of flies to HMIs at mM levels.^21^^,^^26^^,^^27^^,^^31^ In our results, the avoidance response to most of the trace elements increased with concentration and peaked at 2 mM (Figures S1C–S1G). In our test, flies exhibited no preference to Na^+^ at 2 mM, which is beyond the known effective range.^26^ The 0.5 mM Ca^2+^ was capable of inducing a repulsive reaction (Figure S1C), consistent with the previous report.^27^ In contrast, in the process to determine the lower limit of detection, flies could detect Cd^2+^ even at 1 μM (Figure 1C), suggesting that *Drosophila* exhibits a very high sensitivity to these ions.

Flies are known to avoid food containing alkaloids, which are the nitrogenous organic substances with bitter tastes in plants, through bitter-sensitive gustatory receptor neurons (GRNs).^20^ At 0.1 mM, 5 of 7 bitterants (caffeine, CAF; L-canavanine, L-CANA; quinine, QUI; papaverine, PAP; strychnine nitrate salt, STR; lobeline hydrochloride, LOB; denatonium benzoate, DEN) induced obvious repulsive responses (Figure 1D), while at a lower concentration (1 μΜ), only DEN, the most bitter compound to humans and flies, elicited a significant repulsive response (Figures 1D and S1H). Notably, evaluating the strength of avoidance response to Cd^2+^ and DEN at a series of concentrations revealed that at a low concentration, the aversion elicited by Cd^2+^ is comparable in strength to that of the bitterest compound (Figure 1E).

To further investigate whether flies avoid areas containing metal ions, we used a modified positional two-choice assay to quantify the distributions of wild-type flies after feeding with relevant to the media contaminated with metal ions (Figure S2A). The positional responses of sated flies divided the metal ions into three categories with neutral, attractive, and repulsive responses (Figures S2B–S2D), demonstrating the different behavioral valences of these metal ions. Essential trace elements are beneficial for animals at very low levels, but harmful at high concentrations. The elicited behavior characterized here generally correlates with the putative biological effects of these metal ions.

Clustering the metal ions based on their effects in both food-choice and positional-choice assays revealed that flies could distinguish different groups of metal ions and subsequently take diverse actions (Figure 1F). Interestingly, except for Al^3+^ and Ba^2+^, the non-HMIs (Li^+^, Na^+^, K^+^, Ca^2+^, and Mg^2+^) grouped near the control, while the HMIs (Cr^3+^, Mn^2+^, Fe^3+^, Ni^2+^, Cu^2+^, Zn^2+^, Cd^2+^, and Pb^2+^), as well as two strong bitterants (DEN and QUI), were scattered away from the first group. The separation of HMIs from non-HMIs largely reflects the stimulating strength and behavioral valence of HMIs perceived by flies.

The behavioral response to a broad range of metal ions, and hence the ability to sense metal ions, is rather unexpected, given how well *Drosophila* sensory physiology is understood. Further experiments showed that male and female flies avoid substances containing HMIs similarly (Figure S2E). Notably, the ability to detect HMIs is conserved among all four species of *Drosophilidae* tested (Figure S2F), despite several million years of evolutionary distance between them.^32^^,^^33^

Taken together, these behavioral results demonstrate that flies can detect HMIs. The high sensitivity to certain toxic HMIs, such as Cd^2+^, is as good as that to the bitterest compound known. Because Cd^2+^ elicits a strong response, we chose to focus on Cd^2+^ for further investigation to understand the mechanisms of HMIs detection.

### Bitter-sensing neurons detect heavy metal ions

**Figure 2:**
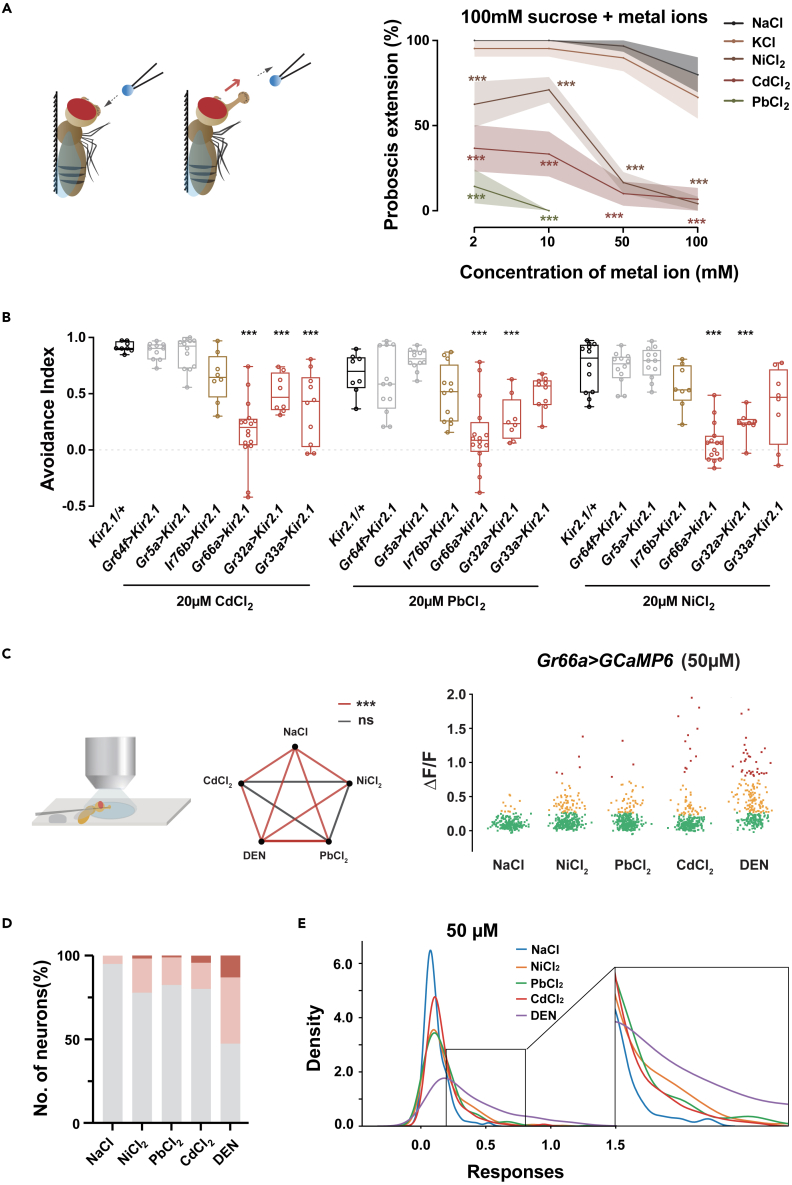
Bitter-taste neurons mediate an aversive response to heavy metals (A) Left: schematic of proboscis extension reflex (PER). A tip containing 100 mM sucrose with metal ions briefly touched the proboscis and withdrew, and extension of the proboscis was monitored. Black dotted arrow indicates the movement of the tip, red arrow indicates the movement of proboscis. Right: quantifying the rate of PER of starved flies to sucrose solution containing different concentrations of metal ions. N = 6–10. (B) Quantifying the avoidance response to HMIs after different taste neurons were specifically silenced. N = 8–16. Gray: sweet-sensing neurons; brown: low salt (NaCl) sensing neurons; red: bitter-sensing neurons. (C) Changes in the fluorescence intensity of GCaMP signals in *Gr66a*^*+*^ neurons when 50 μM of indicated HMIs was applied to the labellum. N = 11–14 flies. Left: Schematic illustrating the calcium imaging preparation. Middle: Statistical analysis of *Gr66a*^*+*^ neurons exposed to 50 μM metal ions and DEN. Na^+^ was the negative control. Right: Analysis of fluorescence changes in labellar *Gr66a*^*+*^ neurons stimulated by metal ions and DEN. Neurons of each stimulation group were clustered into three clusters (K-means, K = 3) and marked with red, orange, or green according to their changes of fluorescent intensity. Genotype: *Gr66a-Gal4/UAS-GCamp6f; +/UAS-tdTomato*. (D) Comparison of proportions of neurons belonging to the three clusters in each stimulation group shown in (C). Gray, pink, and red represent proportions of neurons from green, orange, and red clusters in (C), respectively. (E) Comparing population profile based on the relative change of activities in each stimulation group shown in (C). The distributions of neurons with different activities were fitted with kernel density estimation to generate the population profile. Statistical analyses made comparisons between two groups at the same concentration in (A), one-way ANOVA followed by Tukey’s post hoc test for multiple comparisons among experimental and control group (*UAS-Kir2.1/+*) for each stimulus in (B), and multiple comparisons among experimental and control group (50 μM Na^+^) in (C). ns: p > 0.05, *∗*p < 0.05, *∗∗*p < 0.01, *∗∗∗*p < 0.001. See also Figures S3 and S4.

As flies primarily use their gustatory system to evaluate food before ingestion, we next investigated whether HMIs elicit “bad tastes” that result in food avoidance. In *Drosophila*, taste organs are distributed on the labellum, leg tarsi, wing margins, and pharynx.^7^ We used the proboscis extension reflex (PER) to learn whether the labellum and foreleg could directly sense HMIs. In this assay, added Ni^2+^, Pb^2+^ or Cd^2+^ inhibited PER greatly, and increasing the content of ions resulted in a lower probability of proboscis extension (Figures 2A and S3A–S3C). Direct sensing of HMIs by either the labellum or the foreleg implicates the GRNs on these taste organs in acute HMIs detection. Remarkably, flies with surgically removed tarsi of the legs exhibited an identical level of aversive response to HMIs as wild-type flies (Figure S3D), indicating neurons in the labellum alone are sufficient to mediate strong aversion. Therefore, we focused on the labellum to identify neurons essential for mediating aversive responses.

Next, we tested the behavioral response of flies without functional GRNs. In *Poxn*^*Δm22*^ mutants, poly-innervated chemoreceptors are transformed into mono-innervated mechanosensory receptors during development.^34^ Accordingly, the sensilla on the labellum of *Poxn*^*Δm22*^ mutants appear longer and pointier than that of wild-type flies (Figure S3E). *Poxn*^*Δm22*^ mutants exhibited significantly diminished avoidance of HMIs (Figure S3F), which suggests that GRNs are necessary for HMIs sensation.

GRNs in flies are well-known for being responsible for the detection of different classes of compounds, including sweet compounds (*Gr64f*^*+*^ and *Gr5a*^*+*^ neurons), bitter compounds (*Gr66a*^*+*^, *Gr32a*^*+*^, and *Gr33a*^*+*^ neurons),^20^^,^^35^ salt (*Ir76b*^*+*^ neurons),^26^^,^^31^ amino acids (*Gr66a*^*+*^ neurons, *Ir76b*^*+*^ and *Ir25a*^*+*^ neurons),^25^^,^^36^ fatty acids (*Ir56d*^*+*^ and *Gr64f*^*+*^ neurons),^23^^,^^24^ and acetic acid and lactic acid (*Gr66a*^*+*^ and *Gr64f*^*+*^ neurons).^37^^,^^38^^,^^39^ We set out to identify the GRNs responsible for detecting HMIs, using a selective inhibition approach by overexpressing Kir2.1, an inward-rectifying potassium ion channel, to hyperpolarize the targeted neurons.^40^ Silencing neurons of the bitter-sensing pathway, including *Gr66a*^*+*^, *Gr32a*^*+*^, and *Gr33a*^*+*^ neurons, significantly attenuated the avoidance of Cd^2+^, Pb^2+^, and Ni^2+^, whereas inhibition of other GRNs had minor or no effects (Figure 2B). Our results here expand their ability to a drastically different class of chemicals, HMIs, thereby revealing a new mode of sensation of these neurons.

To investigate whether HMIs trigger activity in GRNs, we performed calcium imaging on gustatory neurons of the labellum using the genetically coded calcium indicator, GCaMP6.^41^^,^^42^ First, we tested the response of *Gr66a*^*+*^ neurons to a bitter compound (DEN) as a positive control to demonstrate the reliability of our system (Figures S4A–S4B). *Gr66a-Gal4* labels all bitter-sensing neurons (approximately 20 neurons) per lobe of the labellum.^35^ NaCl at a concentration of 50 μΜ, 20-fold lower than the low limit for inducing a behavioral response,^26^ was used as a negative control. Although our behavioral paradigm was different, NaCl concentrations 2 mM or lower also failed to elicit clear aversive behaviors (Figure S1C) and when quantifying the activation of individual *Gr66a*^*+*^ neurons with calcium imaging, there was no significant change in fluorescent signals when exposed to 50 μΜ NaCl (Figure S4C). As shown in Figure 2C, Cd^2+^, Ni^2+^, and Pb^2+^, as well as DEN, significantly increased the activity of *Gr66a*^*+*^ neurons. However, responses to HMIs were distinct from those to DEN, but there was no noticeable difference among the three kinds of HMIs (Figure 2C). Based on the strength of these evoked responses, cluster analysis further divided *Gr66a*^*+*^ neurons into three categories with about 6%, 3.7%, and 7.6% of neurons exhibiting high responses to Cd^2+^, Ni^2+^, and Pb^2+^, respectively (Figures 2C, 2D and S4D). Portions of responding neurons (Figure 2D) and the positive skew distribution of evoked signals (Figure 2E) indicate strong heterogeneity of these *Gr66a*^*+*^ neurons in terms of response to HMIs.

Together, these genetic and imaging results support that, in addition to bitterants, HMIs can activate *Gr66a*^*+*^ neurons in the labellum.

### GRNs protect flies from toxic heavy metals ions

**Figure 3:**
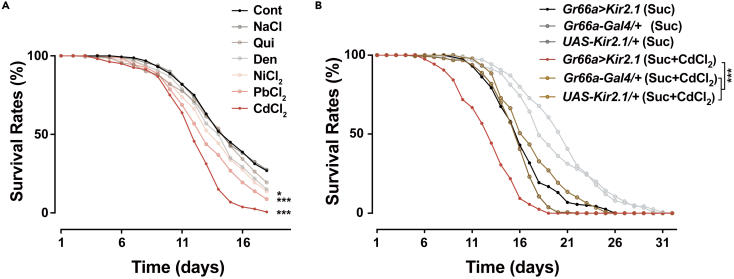
Silencing *Gr66a*^*+*^ neurons aggravates the toxicological effects of heavy metal ions (HMIs) in exposed flies (A) Comparison of survival rates of wild-type flies exposed to the medium containing different HMIs and bitterants (20 μM). Flies in the control group (Cont) were exposed to the medium only. N = 8 groups of 20 flies. (B) Survival of flies with silenced *Gr66a*^*+*^ neurons was strongly decreased when exposed to a medium containing 20 μM CdCl_2_. N = 7–8 groups of 20 flies. The medium in (A) and (B) was 1% agarose with 100 mM sucrose. Statistical analysis: two-way ANOVA for the comparison between the experimental group and genetic controls treated with CdCl_2_ (sucrose +20 μM CdCl_2_). *∗*p < 0.05, *∗∗∗*p < 0.001. See also Figure S4.

The high sensitivity toward HMIs prompted us to determine whether the low concentrations that elicit behavioral aversion would be sufficient to generate physiological effects after prolonged exposure. Chronic exposure to either alkaloids or HMIs severely shortened the lifespan of flies (Figure 3A), demonstrating the dire biotoxicity of HMIs to flies. Compared to Ni^2+^, Pb^2+^, DEN, and QUI, the Cd^2+^-treated group showed even higher toxicity with a median survival at only 15 days. Silencing *Gr66a*^*+*^ neurons aggravated the decrease in lifespan when flies were cultivated on Cd^2+^-containing food (Figure 3B). These results confirm that *Gr66a*^*+*^ neurons also function in HMIs detection and further suggest that the detection sensitivity is physiologically relevant.

We next investigated whether chronic exposure to HMIs modifies the observed aversive response. Five days of pre-exposure to 20 μM Cd^2+^, Pb^2+^, or Ni^2+^ yielded an adaptive effect where flies found the lower concentration of Cd^2+^ more tolerable (Figure S4E), implying a cross-adaptation among these ions. However, food with a concentration of Cd^2+^ similar to or higher than that of pre-exposed Cd^2+^ is still as aversive as before, which indicates that the sensitivity to higher concentrations is maintained despite pre-exposure.

We were also interested in whether the observed shortened lifespan is simply due to a refusal to feed on the contaminated food. We measured the amount of food that remained in the digestive tract after flies were maintained on food containing HMIs for 2 days. Compared with controls, flies treated with HMIs, as well as QUI and DEN, had a decreased amount of food intake but they did not stop feeding (Figure S4F). We speculate that when limited in an environment with water and soil widely polluted, flies are likely left with no choice but to intake food containing HMIs.

### Multiple IRs involved in heavy metal ion sensation in *Gr66a*^*+*^ neurons

In *Gr66a*^*+*^ GRNs, bitter-taste receptors, such as GR32a, GR33a, GR66a, GR89a, and GR93a, participate in the detection of multiple bitter alkaloids.^20^^,^^35^ Therefore, we thought about whether these bitter-taste receptors also participate in detecting HMIs. Notably, all GR mutants tested showed a normal ability to avoid Ni^2+^, Pb^2+^, and Cd^2+^ (Figure S5A). Therefore, gustatory detection of alkaloids and HMIs in *Drosophila* uses distinct signal pathways.

TRP is a family of genes encoding cell surface cation channels.^43^ Mice without TRPV1 or TRPM5 exhibited abnormal perceptions of CuSO_4_, FeSO_4,_ and ZnSO_4_.^17^ In *Drosophila*, TRP channels are known for their vital roles in sensory perception including vision, taste, olfaction, thermosensation, and mechanosensation.^44^ However, all mutants tested exhibited a normal avoidance of Cd^2+^, Pb^2+^, and Ni^2+^ (Figure S5B). Therefore, TRP channels, even those homologous to mammalian *TRPV1* (*TrpA1* and *pain*) and *TRPM5* (*Trpm*), are not required for HMIs sensation.

**Figure 4:**
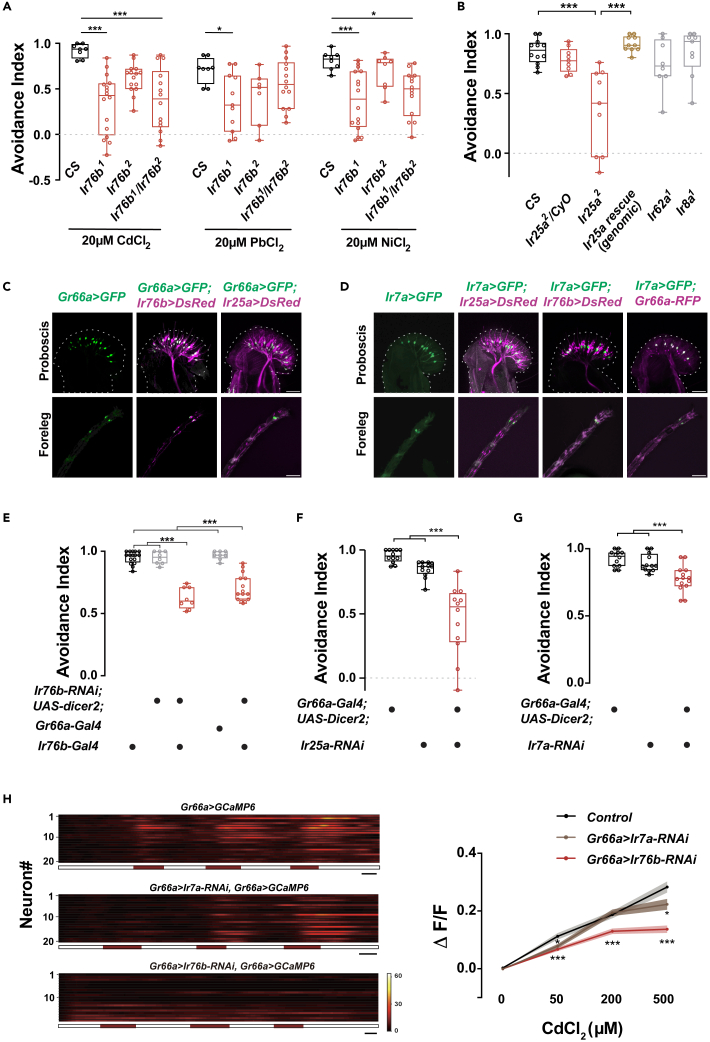
IR76b, IR25a, and IR7a in *Gr66a*^*+*^ neurons regulate Cd^2+^ aversion (A) Comparison of the feeding avoidance responses of *Ir76b* mutants and wild-type flies to the medium containing Cd^2+^, Pb^2+^, or Ni^2+^. N = 8–16. (B) Behavioral responses of *Ir25a* mutants (heterozygous, homozygous, and genomic rescue), *Ir62a* mutants, and *Ir8a* mutants to 20 μM Cd^2+^ in food-choice tests. N = 8–12. (C) Co-expression of *Gr66a*^*+*^ neurons (green) and *Ir76b*^*+*^ or *Ir25a*^*+*^ neurons (magenta) in the labellum (top) and tarsi of forelegs (bottom). Genotypes: *UAS-GFP/+; Gr66a-Gal4/+* (left), *Ir76b-QF/UAS-GFP; Gr66a-Gal4/QUAS-mtdTomato* (middle), and *LexAop-DsRed/+; Ir25a-LexA/+; UAS-GFP/Gr66a-Gal4* (right). (D) Co-expression of *Ir7a*^*+*^ neurons (green) and *Ir76b*^*+*^ neurons, *Ir25a*^*+*^ neurons or *Gr66a*^*+*^ neurons (magenta) in the labellum (top) and foreleg tarsi (bottom). Genotypes from left to right: *Ir7a-Gal4/+; UAS-GFP/+* (1), *LexAop-DsRed/+; Ir7a-Gal4/Ir25a-LexA; UAS-GFP/+* (2), *Ir76b-QF/UAS-GFP; Ir7a-Gal4/QUAS-mtdTomato* (3), and *Gr66a-RFP/UAS-GFP; Ir7a-Gal4/Gr66a-RFP* (4). (E) Behavioral responses to 20 μM Cd^2+^ with *Ir76b* knockdown via RNAi in *Gr66a*^*+*^ or *Ir76b*^*+*^ neurons. N = 8–16. (F) Behavioral responses to 20 μM Cd^2+^ with *Ir25a* knockdown specifically in *Gr66a*^*+*^ neurons. N = 12. (G) Behavioral responses to 20 μM Cd^2+^ with the tuning receptor *Ir7a* knocked down specifically in *Gr66a*^*+*^ neurons. N = 12–14. (H) Analysis of fluorescence changes in labellar *Gr66a*^*+*^ neurons when stimulated by Cd^2+^. Left: visualizing the responses of all *Gr66a*^*+*^ neurons in a single fly of different genotypes. The horizontal bar below the traces indicates the periods of Cd^2+^ application (50 μM, 200 μM or 500 μM Cd^2+^). Scale bar: 2 min. Right: comparison of average fluorescent changes between the control and *Ir7a-RNAi* or *Ir76b-RNAi* flies. Data are represented as mean ± SEM. N = 12. The genotype of control: *Gr66a-Gal4/+; UAS-GCaMP6f, UAS-mtdTomato/+*. Genotypes of the experimental group: *Gr66a-Gal4/UAS-Ir7a-RNAi; UAS-GCaMP6f, UAS-mtdTomato/UAS-dicer2* and *Gr66a-Gal4/UAS-Ir76b-RNAi; UAS-GCaMP6f, UAS-mtdTomato/UAS-dicer2*. The outline of the labellum in (C) and (D) is traced by a dotted line. Scale bar: 50 μm. One-way ANOVA followed by Tukey’s post hoc test for multiple comparisons in (A, B and E–G). In (H), Student’s *t* test was used for comparisons between two groups at the same concentration. *∗*p < 0.05, *∗∗∗*p < 0.001. See also Figure S5.

We extended the scope of our screen to include ion channels, from which we identified IR76b, an ionotropic co-receptor broadly expressed in the gustatory system (Figure S5C). Compared with wild-type flies, *Ir76b* mutants showed significantly decreased avoidance of Cd^2+^ (Figure 4A). Testing over a series of concentrations of Cd^2+^ revealed that the aversive response of *Ir76b*^*1*^ was much weaker than that of wild-type flies across the range, with a stronger difference at the lowest concentration (Figure S5D). This suggests that *Ir76b* is necessary for the normal detection of HMIs, while an additional aversive response, probably independent of *Ir76b*, is triggered as the concentration of Cd^2+^ is elevated.

Another ionotropic co-receptor broadly expressed in the gustatory system is IR25a, which is often found to function together with IR76b.^45^ *Ir25a* and *Ir76b* were co-expressed in labellar neurons.^23^ The reduced avoidance response of *Ir25a* mutants and restored avoidance response of *Ir25a* genomic rescue flies toward Cd^2+^ indicates that *Ir25a* is required to detect HMIs (Figure 4B). As a negative control, the mutants of another co-receptor expressed in the olfactory system but not in the gustatory system, *Ir8a*,^45^ exhibited normal avoidance responses (Figure 4B). Furthermore, IR62a,^27^ a critical tuning receptor for Ca^2+^-elicited aversive responses, was irrelevant to Cd^2+^ sensation (Figure 4B). These results suggest that in addition to the difference in detection limit, gustatory sensing of Ca^2+^ and Cd^2+^ involves different mechanisms.

IR76b and IR25a were present in a portion of *Gr66a*^*+*^ neurons in the labellum (Figure 4C). Knockdown with *Ir76b* RNAi in either *Ir76b*^*+*^ or *Gr66a*^*+*^ neurons rendered a reduced avoidance of Cd^2+^ (Figure 4E). Expressing *Ir76b* in *Ir76b*^*+*^ neurons, or even in *Gr66a*^*+*^ neurons restored the avoidance response of the *Ir76b*^*1*^ mutant (Figure S5E). Additionally, reducing the expression of *Ir25a* in *Gr66a*^*+*^ neurons with RNAi also diminished avoidance performance (Figure 4F). Notably, when the mutants of *Ir76b* and *Ir25a* were tested for the avoidance response to DEN, their performance was similar to that of wild-type controls (Figure S5F), demonstrating that *Ir76b* and *Ir25a* specifically mediate HMIs sensation. Combined with the result that the bitterants receptors are not required for HMIs detection, behavioral avoidance of HMIs and alkaloids is likely based on distinct sensory pathways in *Gr66a*^*+*^ neurons.

IR76b and IR25a are the broadly expressed co-receptors, believed to exert specific functions by forming heteromeric complexes with sparsely expressed tuning receptors.^22^^,^^46^^,^^47^^,^^48^^,^^49^^,^^50^ To identify the tuning receptors for HMIs detection, we screened members of the IR family expressed in *Gr66a*^*+*^ neurons and found that knockdown of *Ir7a* in *Gr66a*^*+*^ neurons reduced the Cd^2+^, Pb^2+^, and Ni^2+^ avoidance response (Figures 4D, 4G, and S5H). This indicates that these IRs participate in the detection of multiple HMIs. *Ir7a* was reported to be required for the avoidance of acetic acid in a subset of *Gr66a*^*+*^ neurons,^38^ it appears that *Ir7a* is at the joint pathways mediating the detection of acetic acid and HMIs, both of which are aversive cues.

To investigate the functional role of multiple IRs in HMIs detection, we directly measured the neuronal activity of *Gr66a*^*+*^ neurons, with a normal or reduced expression level of *Ir76b or Ir7a*, when stimulated with HMIs. The response profiles revealed that a subpopulation of *Gr66a*^*+*^ neurons was activated by Cd^2+^ (Figure 4H). Notably, when the *Ir76b* or *Ir7a* expression was reduced by RNAi, neuronal activation was diminished (Figure 4H). The results demonstrate that HMIs activate *Gr66a*^*+*^ neurons, and this process requires *Ir76b* and *Ir7a*.

Taken together, IR76b, IR25a, and IR7a in *Gr66a*^*+*^ neurons of the labellum play a central role in acute sensation and avoidance of Cd^2+^.

### IR47a-mediated heavy metal ion sensation in non-*Gr66a*^*+*^ neurons

The reminiscent avoidance of Cd^2+^ in *Gr66a>Kir2.1* flies (Figure 2B) implies that while all *Gr66a*^*+*^ neurons are silenced, additional neurons help flies detect HMIs. Because IRs are considered another repertoire of gustation in addition to GRs, we addressed whether these unidentified neurons use IRs for HMIs sensing. We screened through *Ir-Gal4* labeled neurons for their essential roles in the behavioral avoidance of Cd^2+^ and identified *Ir47a-Gal4* labeled neurons (Figure S6A). *Ir47a*^*+*^ neurons were located on the labellum and legs (Figure S6C), consistent with a previous report.^45^

**Figure 5:**
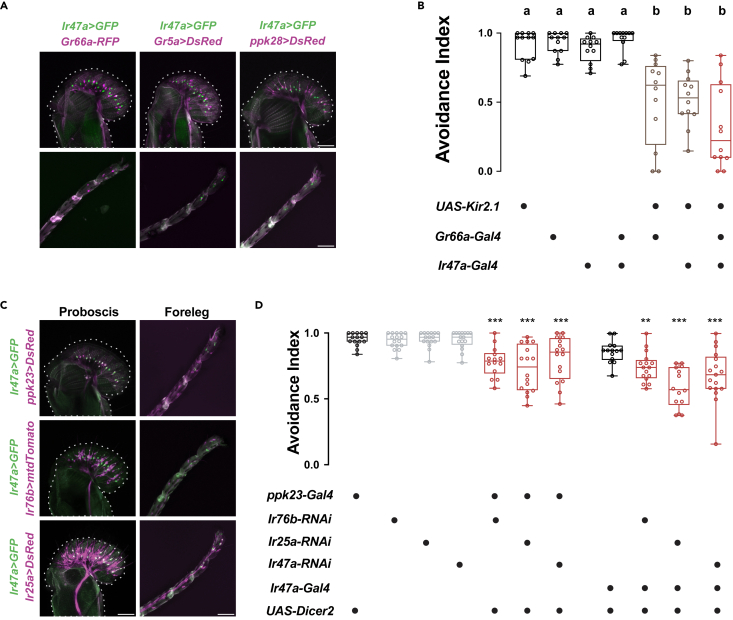
The Cd^2+^ sensing *Ir47a*^*+*^ neurons are *Gr66a*-negative but *ppk23*-positive (A) Co-expression of *Ir47a*^*+*^ neurons (green) and *Gr66a*^*+*^ neurons, *Gr5a*^*+*^ neurons, *ppk28*^*+*^ neurons (magenta) in the labellum (top) and tarsi (bottom). Genotypes from left to right*: Gr66a-RFP/UAS-GFP; Ir47a-Gal4/Gr66a-RFP* (1), *LexAop-DsRed/+; Gr5a-LexA/UAS-GFP; Ir47a-Gal4/+* (2), *LexAop-DsRed/+; +/UAS-GFP; Ir47a-Gal4/ppk28-LexA* (3). (B) Evaluating the combined effects of *Ir47a*^*+*^ neurons with *Gr66a*^*+*^ neurons on avoidance of 20 μM Cd^2+^. N = 12. (C) Co-expression of *Ir47a*^*+*^ neurons (green) and *ppk23*^*+*^ neurons, *Ir76b*^*+*^ neurons, or *Ir25a*^*+*^ neurons (magenta) in the labellum (left) and tarsi (right). Genotypes: *LexAop-DsRed/+; ppk23-LexA/UAS-GFP; Ir47a-Gal4/+* (top), *Ir76b-QF/UAS-GFP; Ir47a-Gal4/QUAS-mtdTomato* (middle), and *LexAop-DsRed/+; Ir47a-Gal4/Ir25a-LexA; UAS-GFP/+* (bottom). (D) Avoidance responses of flies with *Ir76b*, *Ir25a*, or *Ir47a* knocked down in *ppk23*^*+*^ or *Ir47*^*+*^ neurons via RNA interference to 20 μM Cd^2+^. N = 14–16. Scale bar: 50 μm. One-way ANOVA followed by Tukey’s post hoc test for multiple comparisons in (B) and (D). In (B), a and b indicate the multiple comparison results among various columns. The same letters indicate no significant difference; different letters indicate statistically significant differences. *∗∗*p < 0.01. *∗∗∗*p < 0.001. See also Figure S6.

GRNs in the fly labellum are grouped into five classes: A neurons (sweet), B neurons (bitter), C neurons (water), D neurons (expressing in *ppk23*^*glut*^ neurons), and E neurons (expressing IR94e).^51^ In terms of behavioral valence, A, C, and E neurons mediate attraction responses, while B and D neurons mediate rejection responses.^51^ We then investigated how *Ir47a*^*+*^ neurons fit into these classes. Co-labeling experiments revealed that *Ir47a*^*+*^ neurons were not coincident with attractive *Gr5a*^*+*^, *ppk28*^*+*^, and *Ir94e*^*+*^ neurons, nor with the repulsive *Gr66a*^*+*^ neurons (Figures 5A and S6D). Although both are required for aversion to Cd^2+^, the neurons labeled by *Ir47a-Gal4* and *Gr66a-Gal4* are two distinct populations. Silencing both populations together resulted in a partial reduction, rather than complete loss, of Cd^2+^ avoidance (Figure 5B), implicating additional pathways mediating Cd^2+^ sensation. Instead, *Ir47a*^*+*^ neurons extensively co-localized with *ppk23*^*+*^ neurons, the repulsive class D neurons (Figure 5C). We further characterized the *Ir47a*^*+*^ neurons for their repertoire of IR co-receptors with co-expression experiments (Figure 5C). The expression patterns of *Ir47a*^*+*^ and *Ir76b*^*+*^ did not fully overlap in Figure 5C, this is likely due to the *QF* reporter of *Ir76b* used, which did not show a pattern fully overlapped with that of the *Gal4* reporter of *Ir76b* (Figure S6E). To circumvent the lack of faithful non-GAL4 *Ir76b* driver, we looked into the labellar neurons known for expressing *Ir76b*, *ppk23*^*+*^ neurons.^28^^,^^51^ As shown in Figure 5C, the extensive co-expression of *Ir47a*^*+*^ and *ppk23*^*+*^ neurons strongly suggest the ubiquitous expression of *Ir76b* in *Ir47a*^*+*^ neurons.

We next analyzed single-cell transcriptome data of the *Drosophila* gustatory system to gain an additional molecular perspective of taste coding of GRNs.^52^ Number of occurrences derived from this dataset served as a qualitative indication, rather quantitative representation, of co-expression frequencies. Expression profiles of *Ir76b*, *Ir25a*, *Ir47a*, and *ppk23* in labellar GRNs of both male and female flies further supported the co-existence of these IRs and ppk23 in the labellum (Figures S6F and S6G).

We next used an RNAi approach to investigate whether the broadly expressed ionotropic co-receptor, IR76b and IR25a, and tuning receptor IR47a are required for *ppk23*^*+*^ neurons to sense HMIs. As shown in Figure 5D, the knockdown of IR76b, IR25a, or IR47a in *ppk23*^*+*^ neurons significantly reduced the avoidance response to Cd^2+^. Similarly, in *Ir47a-Gal4* labeled neurons, decreased expression levels of *Ir76b*, *Ir25a*, or *Ir47a* also reduced the aversive response to Cd^2+^, indicating that all three IRs are needed in *Ir47a*^*+*^ neurons for the proper detection of Cd^2+^ (Figure 5D).

**Figure 6:**
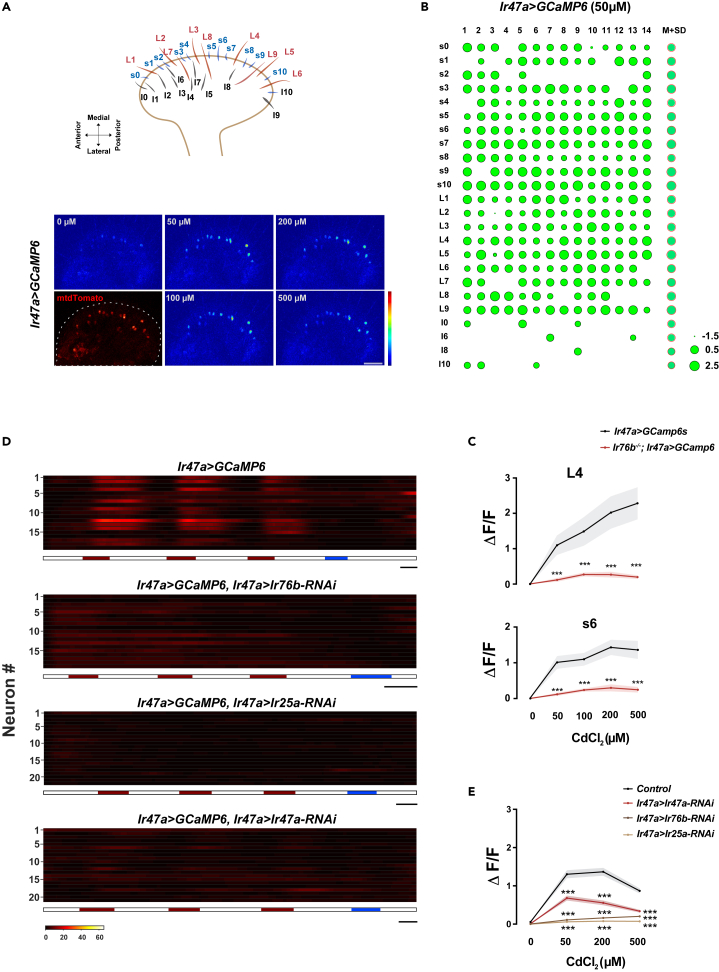
Cd^2+^-induced activation of labellar *Ir47a*^*+*^ neurons requires IR76b, IR25a and IR47a (A) Representative images of Ca^2+^ responses of *Ir47a*^*+*^ neurons before and after sequential stimulation with elevating concentrations of Cd^2+^. The top schematic illustrates the subtypes and locations of chemosensilla on the labellum. The distribution of all *Ir47a*^*+*^ neurons was visualized by *mtdTomato* signals in red channel. Genotype: *Ir47a-Gal4/+; UAS-GCaMP6m, UAS-mtdTomato/+*. Scale bar: 30 μm. (B) Survey of all *Ir47a*^*+*^ neurons in each fly (14 flies) for their response to 50 μM Cd^2+^. The sensilla subtypes of *Ir47a*^*+*^ neurons corresponded to those of sensilla shown in (A). The area of each green circle represents the fluorescent signal intensity of GCaMP. The last column, M + SD, represents the mean value (green) plus one standard deviation (red). (C) Comparing the Ca^2+^ responses of *Ir47a*^*+*^ neurons projecting to sensilla L4 and s6 to Cd^2+^ of different concentrations in wild-type flies and *Ir76b* mutants. N = 12–14. (D and E) Evaluating the activities of all *Ir47a*^*+*^ neurons in single flies when stimulated by Cd^2+^. (D) Example traces. The red bars indicate 50 μM, 200 μM, or 500 μM Cd^2+^ application, and blue bars indicate 2 mM DEN application. From top to bottom: 1. A control fly (*Ir47a-Gal4/+; UAS-GCaMP6m, UAS-tdTomato/+*). 2. A fly with *Ir76b* knockdown (*Ir47a-Gal4/UAS-Ir76b-RNAi; UAS-GCaMP6m, UAS-tdTomato/UAS-dicer2*). 3. A fly with *Ir25a* knockdown (*Ir47a-Gal4/UAS-Ir25a-RNAi; UAS-GCaMP6m, UAS-tdTomato/UAS-dicer2*). 4. A fly with *Ir47a* knockdown (*Ir47a-Gal4/UAS-Ir47a-RNAi; UAS-GCaMP6m, UAS-tdTomato/UAS-dicer2*). Scale bar: 2 min. (E) Quantification of the fluorescence changes elicited by different concentrations of Cd^2+^ in flies with decreased expression of *Ir76b, Ir25a*, or *Ir47a* in *Ir47a*^*+*^ neurons. Data are represented as mean ± SEM. N = 12–13 flies. Student’s *t* test for comparisons between two groups at the same concentration in (C) and (E). *Z* score normalization of fluorescent data from each fly was performed in (B)–(C). *∗∗∗*p < 0.001. See also Figure S6.

*Ir47a*^*+*^ neurons project into almost all L-type and s-type sensilla in the labellum.^48^ To survey the excitability of the *Ir47a*^*+*^ population induced by Cd^2+^, we visualized the activity states of all *Ir47a*^*+*^ neurons with *ex vivo* calcium imaging.^42^ Each *Ir47a*^*+*^ neuron projects to different sensilla. All *Ir47a*^*+*^ neurons responded strongly to Cd^2+^ (Figures 6A–6C), demonstrating that non-*Gr66a*^*+*^ neurons mediate aversive reactions to HMIs in addition to *Gr66a*^*+*^ neurons. Besides L-type and s-type sensilla, we occasionally observed that *Ir47a*^*+*^ neurons projecting to I-type sensilla were activated by Cd^2+^ (Figure 6B). We chose neurons projecting to L4 and s6 sensilla for further analysis. When removing *Ir76b* from these neurons, the responses of L4 and s6 sensilla to Cd^2+^ were severely affected (Figure 6C). Additionally, calcium imaging results indicate that consistent with the lack of *Ir47a* expression in *Gr66a*^*+*^ neurons, *Ir47a*^*+*^ neurons were not excited by DEN (Figure 6D, blue mark). Moreover, the lack of neuronal responses of *Ir47a*^*+*^ neurons to Cd^2+^ when *Ir76b, Ir25a*, or *Ir47a* was knocked down by RNAi suggests that both IR76b, IR25a, and IR47a are required in *Ir47a*^*+*^ neurons to detect Cd^2+^ (Figures 6D and 6E).

To evaluate the detection spectrum of *Ir47a*^*+*^ neurons, we analyzed whether other HMIs could elicit an aversive behavior in flies when *Ir47a*^*+*^ neurons were silenced. In addition to Cd^2+^, Pb^2+^ was also avoided when *Ir47a*^*+*^ neurons were silenced by Kir2.1 (Figure S6H). Decreasing the expression of *Ir47a* in *Ir47a*^*+*^ neurons also reduced the avoidance responses to Pb^2+^ and Ni^2+^, supporting a general role of *Ir47a* in the sensation of HMIs (Figure S6I).

Taken together, taste perception provides flies with the capability to avoid food contaminated by HMIs. This is accomplished through two parallel pathways in the gustatory system, both of which are mediated by IRs. IR76b, IR25a, and IR7a confer upon the typical *Gr66a*^*+*^ neurons a new taste modality to sense HMIs, while in a distinct set of gustatory neurons, IR47a, together with IR76b and IR25a, contributes to the avoidance of noxious HMIs.

## Discussion

We found that *Drosophila* exhibit a robust avoidance response to HMIs, especially Cd^2+^. The high sensitivity to Cd^2+^ is comparable to that of denatonium. HMIs activate *Gr66a*^*+*^ neurons, but the canonical bitter-taste receptors are dispensable for this process. From several screens, we identified IR76b, IR25a, and IR7a are required for sensing HMIs in *Gr66a*^*+*^ neurons, and a group of GR66a-independent neurons, which require IR76b, IR25a, and IR47a are also mediating HMIs-induced aversion. These findings offer a vital framework for understanding the biological detection of toxic HMIs.

The unexpected sensitivity and breadth of the tuning of a gustatory response elicited by a class of agents with similar chemical natures suggest an additional taste quality or taste category. The uniformity of underlying molecules for sensing HMIs, the IRs, further strengthens the qualitative attribute. In this respect, *Drosophila* is not alone. Throughout the history of the psychophysics of human taste, the metallic taste has been proposed as a basic taste, along with sweet, bitter, sour, and salty.^53^ It is difficult to correlate the complex taste experience in humans with taste-evoked behavior in animals. Nevertheless, the response of the human bitter receptor TAS2R7 to multiple HMIs offers interesting parallelism to our finding in *Drosophila*.

It is also equally important for animals not to reject all HMIs outright because some are essential for survival. Particularly, trace elements are required for a variety of biological processes,^2^ and thus they are beneficial at a low level. However, at high levels, they interfere with physiological functions. The complex characteristics and biological functions of HMIs likely contribute to the heterogeneity of behavioral responses in *Drosophila*. Although our results were consistent with the recent findings of feeding avoidance induced by essential trace elements at millimole concentrations,^28^^,^^29^ we found that some essential trace elements at lower concentrations elicit the opposite behavioral response. As such, in the positional choices assay with 0.1 mM HMIs, flies are attracted to Cr^2+^, Fe^3+^, Zn^2+^, and Al^3+^ and are not repelled by Li^+^, Na^+^, K^+^, Ca^2+^, Mg^2+^, and Ba^2+^. The aversion to Cd^2+^ and Pb^2+^ even at much lower concentrations is consistent with their toxic nature in a broad concentration range. It is not immediately clear why Ni^2+^, which is an essential trace element, elicits a strong aversive response, though this may represent a *Drosophila*-specific trait. It is worth noting that previous investigations conducted in worms, rodents, and humans covered only Ca, Mg, Fe, Cu, and Zn, at comparatively higher concentrations. The taste sensitivity and taste quality of Cd and other toxic HMIs in organisms beyond *Drosophila* remain to be determined. Furthermore, the protective role of a taste system needs to be established by comparing the detection limit and the minimum toxic level of HMIs.

The gustatory system of *Drosophila* has served as a major model system for investigations on taste. For each taste modality, different types of taste-responding neurons harbor a combination of cell surface receptors for chemical stimulants.^19^^,^^20^ Our finding that *Gr66a*^*+*^ neurons are also activated by HMIs to trigger avoidance behavior expands their response profiles and confirms the general role of *Gr66a*^*+*^ neurons in warning animals of toxic agents.^54^^,^^55^ However, *Gr66a*^*+*^ neurons use distinct sensing mechanisms for HMIs and bitter agents. These findings are consistent with the emerging concept that bitter-taste neurons are heterogeneous in terms of receptor repertoires and functions.^56^^,^^57^ Moreover, the perceptions of alkaloids and HMIs are likely still distinguishable because of the additional contributions from GR66a-independent neurons sensitive to HMIs. Two classes of GRNs in the labellum, B (bitter) and D (*ppk23*^*glut*^ neurons) neurons, mediate rejection responses.^51^ Our findings here expand the response profiles of *ppk23*^*+*^ neurons to include toxic HMIs. Specifically, *ppk23*^*+*^ neurons in the labellum were involved in the sensation of high salt, Ca^2+^, and Zn^2+^ using IR76b and IR25a as co-receptors, while specificity was determined by tuning IRs.^27^^,^^28^^,^^51^ Based on their neurotransmitters, *ppk23*^*+*^ neurons can be further divided into *ppk23*^*chat*^ neurons and *ppk23*^*glut*^ neurons. While *ppk23*^*glut*^ neurons are negative for GR66a, *ppk23*^*chat*^ neurons express GR66a.^51^ Based on our co-labeling experiment, we conclude that *Ir47a*^*+*^ neurons do not show any expression of GR66a. This suggests that these sensing neurons belong to the *ppk23*^*glut*^ subset, rather than the *ppk23*^*chat*^ subset, of class D neurons. The fact that *Drosophila*, and potentially other *Drosophilidae*, utilizes both B and D classes of GRNs in Cd^2+^ sensation signifies the critical importance of active avoidance of HMIs for survival.

Both B and D classes of GRNs drive high salt avoidance as well.^51^ In the B class GRNs, the molecular mechanism of high salt sensation is unclear, although IR76b is required for this function.^51^ This suggests that high salt and HMI activate different taste signal pathways in this class. On the other hand, in the D class, IR 7c (IR7c) functioning with co-receptors IR76b and IR25a was found to detect high salt.^58^^,^^59^ Although both high salt and HMI require co-receptors IR76b and IR25a in the D class, the distinct tuning receptors would allow flies to distinguish between these stimuli. Furthermore, within the D class, the responsive neurons for high salt and HMI are likely different with high salt activating the *Ir7c*^*+*^ *ppk23*^*glut*^ neurons, whereas *Ir47a*^*+*^ neurons respond strongly to Cd^2+^. The presence of additional HMI-sensitive *ppk23*^*glut*^ neurons might also lead to distinct tastes of high salt and HMIs. Even if IR47a is also involved in sensation to high salt (≥500 mM), one needs to explain why such a low sensitivity to Na^+^ is not a nonspecific response as the sensitivity to Cd^2+^ is below1 mM.

The number of HMIs is numerous, with diverse chemical and physiologic properties, it is difficult to expect them as kindred stimuli, ready to be detected by homogeneous sensation mechanisms. Recent studies have shown that GR66a and GR33a are responsible for detecting Cu and Ag (group XI in the periodic table), whereas IR76b and IR25a are responsible for elements in other groups (group XII: Zn and Cd; group X: Ni; group IX: Co; group IIX: Fe; group VIII: Mn).^29^ Although Zn and Cd locate in the same group but in different periods (IV and V, repectively), Zn^2+^ detection is mediated by IR25a, IR76b, and IR56b in *ppk23*^*+*^ neurons.^28^ From the initial screen of seventeen heavy metal elements, we chose to study toxic heavy metals (primarily Cd) as *Drosophila* exhibits extremely high sensitivity to them. Our findings that removing IR47a from *Ir47a*^*+*^ neurons and IR7a from *Gr66a*^*+*^ neurons decreases behavioral aversion to Pb^2+^ and Ni^2+^ suggest that, similar perception mechanisms are shared between certain heavy metals, likely for these with very high atomic numbers and extremely toxic nature.

Different from GRs, IRs belong to a subfamily of ionotropic glutamate receptors, which are ligand-gated ion channels.^22^ IRs have been shown to play diverse roles in olfaction,^22^^,^^60^^,^^61^^,^^62^ taste,^23^^,^^26^ thermosensation,^63^^,^^64^ and hygrosensation.^65^ The co-receptors, IR76b and IR25a, are broadly expressed and facilitate the sensation of both attractive cues (low salt, fatty acid, polyamines, and carbonation) and aversive cues (high salt, Ca^2+^, and Zn^2+^). The tuning IRs are responsible for specificity and valence. Our results suggest that in coordination with IR76b and IR25a, IR7a, and IR47a act as tuning IRs to convey the aversive taste of HMIs in *Gr66a*^*+*^ neurons and GR66a-independent neurons, respectively. Further analysis of the structural-functional relationship of these IR complexes will help reveal novel gating mechanisms leading to HMIs sensation.

Given that sensing and avoiding water and food contaminated by HMIs provides a strong survival benefit to animals, it would not be surprising that multiple organs (proboscis, legs, and pharynx), neurons (*Gr66a*^*+*^ and *Ir47a*^*+*^ neurons), and receptors (IR7a, IR47a, and likely more) work in parallel and at different levels to ensure that fruit flies accomplish the task with sufficient redundancy. We believe that some of the weak phenotypes shown in this and other studies^27^^,^^28^ demonstrate the robustness of such HMIs detection systems against perturbation. Our *Ir*>*Kir2.1* suppression screen hinted that in addition to *Gr66a*^*+*^ and *Ir47a*^*+*^ neurons, other neurons are likely involved in HMIs-induced avoidance. Nevertheless, the roles of labellar *Ir7a*^*+*^ and *Ir47a*^*+*^ neurons are important, as suppressing both neuronal subtypes together resulted in a ∼70% loss in avoidance performance. Redundancy across molecular, cellular, and organ levels is presumably organized into a fail-safe scheme for avoiding naturally occurring toxicity in the complex natural world.

### Limitations of the study

We systematically investigated the neural basis of innate responses in *Drosophila* to seventeen different metal ions from a broad perspective. Our findings suggest that distinct IRs repertoire in parallel taste transduction pathways mediate high sensitivity to toxic HMIs. However, our research has a few limitations that could be addressed as follows. First, besides silencing the target neurons, the RNAi technique was also utilized as a primary method to disrupt the functions of these neurons by reducing the expression levels of key GRs and IRs. Conducting mutant, rescue and ectopic experiments of IR47a and IR7a could provide further evidence for the involvement of IR repertoire in the perception of HMIs. Secondly, based on their responses to Cd^2+^, *Gr66a*^*+*^ neurons are divided into three clusters: strong responders, weak responders, and non-responders, and *Ir7a*, which is expressed only in a subset of *Gr66a*^*+*^ neurons, could help to divide the *Gr66a*^*+*^ population for further understanding the molecular nature of their diverse response. Thirdly, our imaging results suggest that all *Ir47a*^*+*^ neurons responded to Cd^2+^. *Ir47a*^*+*^ neurons constitute about 90% *ppk23*^*+*^ population in the labellum. This adds another dimension to the response profile of *ppk23*^*+*^ neurons, which are well-known for high salt sensation. Our results, together with the recent discovery that Ca^2+^ and Zn^2+^ are detected by the *ppk23*^*+*^ population with different IR repertoires, indicate a need for in-depth dissection of the *ppk23*^*glut*^ population for their specific roles and sensitivities in the perception of Na^+^, Ca^2+^, Zn^2+^. and Cd^2+^. Lastly, as both GRs and IRs are involved in HMI detection, the combination codes of these receptors possibly determine the specificity toward individual metal ions. Further investigation via calcium imaging of GRN with multi-RNAi knockdown could provide a broader understanding of the response profile of different ions at both the molecular (GRs/IRs repertoire) and neuronal (subsets of GRNs) levels.

## STAR★Methods

### Key resources table

**Table:** 

REAGENT or RESOURCE	SOURCE	IDENTIFIER
**Chemicals, peptides, and recombinant proteins**		

NaCl	Sinopharm Chemistry	10019308
CaCl_2_	Sinopharm Chemistry	10005861
FeCl_3_·6H_2_O	Sinopharm Chemistry	10011918
KCl	Xilong Sientific	10200501
MgCl_2_·6H_2_O	Xilong Sientific	10500301
BaCl_2_	Xilong Sientific	12100301
AlCl_3_	Xilong Sientific	11200801
ZnCl_2_	SIGMA	Z0152
CuCl_2_	ALDRICH	222011
Co (NO_3_)_2_	ALDRICH	2367
Er (NO3)_2_	Adamas	10187A
MnCl_2_·4H_2_O	Xilong Sientific	1040101
LiCl	Xilong Sientific	11000101
NiCl_2_	ALDRICH	339350
CdCl_2_	ALDRICH	529575
CrCl_3_	ALDRICH	230723
PbCl_2_	MACKUN	L812466
Caffeine	SIGMA	P3510
Lobeline hydrochloride	ALDRICH	141879
Strychnine hemisulfate salt	SIGMA	S7001
Quinine hydrochloride dihydrate	TCI	Q0030
L-canavanine sulfate salt	SIGMA	C9758
Denatonium benzoate	SIGMA	D5765
Papaverine hydrochloride	SIGMA	P3510
Sucrose	SIGMA	S0389
Brilliant Blue FCF	Fluka	80717
Sulforhodamine B	SIGMA	341738
Erioglaucine Disodium Salt	Sigma	861146
Glycerol	Xilong Scientific	1281801
Tween-20	Lab EAD	P1379

**Experimental models: Organisms/strains**		

*Drosophila: Canton S*	(Zhan et al.^66^)	N/A
*Drosophila melanogaster*	Core Facility of Drosophila Resource and Technology, Chinese Academy of Scieces	N/A
*Drosophila simulans*	Core Facility of Drosophila Resource and Technology, Chinese Academy of Scieces	N/A
*Drosophila sechellia*	Core Facility of Drosophila Resource and Technology, Chinese Academy of Scieces	N/A
*Drosophila yakuba*	Core Facility of Drosophila Resource and Technology, Chinese Academy of Scieces	N/A
*Drosophila: Gr64f-Gal4*	John Carlson lab, Yale University	N/A
*Drosophila: Gr5a-Gal4*	Kristin Scott lab, University of California, Berkeley	N/A
*Drosophila: Ir76b-Gal4*	Bloomington Drosophila Stock Center	RRID: BDSC51311
*Drosophila: Gr66a-Gal4*	Bloomington Drosophila Stock Center	RRID: BDSC57670
*Drosophila: Gr32a-Gal4*	John Carlson lab, Yale University	N/A
*Drosophila: Gr33a-Gal4*	Bloomington Drosophila Stock Center	RRID: BDSC31425
*Drosophila: UAS-Kir2.1*	Bloomington Drosophila Stock Center	RRID: BDSC6595
*Drosophila: Poxn*^*Δm22*^	Yi Rao lab, Peking University	N/A
*Drosophila: Ir76b*^*1*^	Bloomington Drosophila Stock Center	RRID: BDSC51309
*Drosophila: Ir76b*^*2*^	Bloomington Drosophila Stock Center	RRID: BDSC51310
*Drosophila: UAS-mCD8::GFP*	(Zhan et al.^66^)	N/A
*Drosophila: Ir76b-QF*	Bloomington Drosophila Stock Center	RRID: BDSC51312
*Drosophila: QUAS-mtdTomato*	Bloomington Drosophila Stock Center	RRID: BDSC30005
*Drosophila: UAS-Ir76b*	Bloomington Drosophila Stock Center	RRID: BDSC52610
*Drosophila: UAS-Ir76b-RNAi*	Bloomington Drosophila Stock Center	RRID: BDSC54846
*Drosophila: UAS-Dcr2*	Jose Pastor lab, Tsinghua University	N/A
*Drosophila: UAS-GCaMP6m, UAS-tdTomato*	Wei Zhang lab, Tsinghua University	N/A
*Drosophila: Gr66a*^*ex83*^	Bloomington Drosophila Stock Center	RRID: BDSC28804
*Drosophila: Gr33a*^*1*^	Bloomington Drosophila Stock Center	RRID: BDSC31427
*Drosophila: ▵Gr32a*	Craig Montell lab, University of California Santa Barbara	N/A
*Drosophila: Gr93a*^*1*^	Bloomington Drosophila Stock Center	RRID: BDSC18458
*Drosophila: TrpA1*^*1*^	Bloomington Drosophila Stock Center	RRID: BDSC26504
*Drosophila: pain*^*1*^	Bloomington Drosophila Stock Center	RRID: BDSC27895
*Drosophila: wtrw*^*1*^	Craig Montell lab, University of California Santa Barbara	N/A
*Drosophila: pyx*^*3*^	Craig Montell lab, University of California Santa Barbara	N/A
*Drosophila: Trpl*^*MB10553*^	Bloomington Drosophila Stock Center	RRID: BDSC29134
*Drosophila: Trpγ*^*MB06664*^	Bloomington Drosophila Stock Center	RRID: BDSC25629
*Drosophila: Trp*^*MB03672*^	Bloomington Drosophila Stock Center	RRID: BDSC23636
*Drosophila: Trpml*^*1*^	Bloomington Drosophila Stock Center	RRID: BDSC28992
*Drosophila: trpm*^*2*^	Yi Rao lab, Peking University	N/A
*Drosophila: NompC3*	Y.N. Jan lab, University of California, San Francisco	N/A
*Drosophila: iav*^*1*^	Yi Rao lab, Peking University	N/A
*Drosophila: nan*^*36*^	Bloomington Drosophila Stock Center	RRID: BDSC24902
*Drosophila: amo*^*1*^	Craig Montell lab, University of California Santa Barbara	N/A
*Drosophila: Ir25a-Gal4*	Bloomington Drosophila Stock Center	RRID: BDSC41728
*Drosophila: Gr66a-RFP*	Bloomington Drosophila Stock Center	RRID: BDSC60691
*Drosophila: Ir25a*^*2*^	Bloomington Drosophila Stock Center	RRID: BDSC41737
*Drosophila: Ir25a*^*rescue*^	Bloomington Drosophila Stock Center	RRID: BDSC78068
*Drosophila: Ir8a*^*1*^	Bloomington Drosophila Stock Center	RRID: BDSC41744
*Drosophila: Ir62a*^*1*^	Bloomington Drosophila Stock Center	RRID: BDSC32713
*Drosophila: UAS-Ir25a-RNAi*	Bloomington Drosophila Stock Center	RRID: BDSC43985
*Drosophila: UAS-Ir7a-RNAi*	Vienna Drosophila Resource Center	RRID: v108171
*Drosophila: Ir47a-Gal4*	Bloomington Drosophila Stock Center	RRID: BDSC60696
*Drosophila: Ir47a (2)-Gal4*	Bloomington Drosophila Stock Center	RRID: BDSC60695
*Drosophila: UAS-Ir47a-RNAi*	Vienna Drosophila Resource Center	RRID: v11812
*Drosophila: Ir7a-Gal4*	Bloomington Drosophila Stock Center	RRID: BDSC41741
*Drosophila: Ir7b-Gal4*	Bloomington Drosophila Stock Center	RRID: BDSC81219
*Drosophila: Ir7c-Gal4*	Bloomington Drosophila Stock Center	RRID: BDSC81623
*Drosophila: Ir10a-Gal4*	Bloomington Drosophila Stock Center	RRID: BDSC81224
*Drosophila: Ir11a-Gal4*	Bloomington Drosophila Stock Center	RRID: BDSC41742
*Drosophila: Ir20a-Gal4*	Bloomington Drosophila Stock Center	RRID: BDSC60693
*Drosophila: Ir52a-Gal4*	Bloomington Drosophila Stock Center	RRID: BDSC81229
*Drosophila: Ir52b-Gal4*	Bloomington Drosophila Stock Center	RRID: BDSC81230
*Drosophila: Ir52c-Gal4*	Bloomington Drosophila Stock Center	RRID: BDSC60701
*Drosophila: Ir56a-Gal4*	Bloomington Drosophila Stock Center	RRID: BDSC81233
*Drosophila: Ir56b-Gal4*	Bloomington Drosophila Stock Center	RRID: BDSC60706
*Drosophila: Ir56d-Gal4*	Bloomington Drosophila Stock Center	RRID: BDSC60708
*Drosophila: Ir60b-Gal4*	Bloomington Drosophila Stock Center	RRID: BDSC81627
*Drosophila: Ir60c-Gal4*	Bloomington Drosophila Stock Center	RRID: BDSC81628
*Drosophila: Ir60d-Gal4*	Bloomington Drosophila Stock Center	RRID: BDSC81629
*Drosophila: Ir62a-Gal4*	Bloomington Drosophila Stock Center	RRID: BDSC60713
*Drosophila: Ir67c-Gal4*	Bloomington Drosophila Stock Center	RRID: BDSC81239
*Drosophila: Ir94a-Gal4*	Bloomington Drosophila Stock Center	RRID: BDSC60720
*Drosophila: Ir94b-Gal4*	Bloomington Drosophila Stock Center	RRID: BDSC81631
*Drosophila: Ir94c-Gal4*	Bloomington Drosophila Stock Center	RRID: BDSC60721
*Drosophila: Ir94e-Gal4*	Bloomington Drosophila Stock Center	RRID: BDSC81246
*Drosophila: Ir94f-Gal4*	Bloomington Drosophila Stock Center	RRID: BDSC60726
*Drosophila: Ir94h-Gal4*	Bloomington Drosophila Stock Center	RRID: BDSC60728
*Drosophila: Ir100a-Gal4*	Bloomington Drosophila Stock Center	RRID: BDSC41743
*Drosophila: Gr5a-LexA*	Kristin Scott lab, University of California, Berkeley	N/A
*Drosophila: ppk23-LexA*	Kristin Scott lab, University of California, Berkeley	N/A
*Drosophila: ppk28-LexA*	Bloomington Drosophila Stock Center	RRID: BDSC93022
*Drosophila: ppk23-Gal4*	Wei Zhang lab, Tsinghua University	N/A
*Drosophila: Ir25a-LexA*	Wei Zhang lab, Tsinghua University	N/A
*Drosophila: LexAOP-DeRed*	Junhai Han lab, Southeast University	N/A
*Drosophila: UAS-TNT*	Aike Guo and Yan Li lab, Institute of Biophysics, Chinese Academy of Sciences	N/A

**Software and algorithms**		

Prism 8	GraphPad Prism https://www.graphpad.com/	RRID:SCR_002798
MATLAB 2018a	MathWorks, https://www.mathworks.com/products/matlab.html	RRID:SCR_006752
Fiji	NIH https://fiji.sc/	RRID:SCR_002285
Adobe Illustrator	Adobe https://www.adobe.com/	RRID:SCR_010279

**Deposited data**		

Raw and analyzed data	This paper; Mendeley Data	Menledey Data: https://doi.org/10.17632/9dpmj77y4f.1

### Resource availability

#### Lead contact

Further information and requests for resources and reagents should be directed to and will be fulfilled by the lead contact, Yan Zhu (zhuyan@ibp.ac.cn).

#### Materials availability

All *Drosophila* strains are available from the lead contact.

### Experimental model and study participant details

#### Fly stocks and chemicals

Flies were raised on standard food at 25°C or 29°C (RNAi lines), with a humidity of 60%. Details of *Drosophila* strains and chemicals used are in the key resources table. *Canton-S* was used as wild-type strain.

### Method details

#### Two-choice feeding assay

Two-choice feeding assay was modified from a standard protocol.^67^ Thirty 3–5-day-old female flies were first kept in a vial containing only filter paper soaked with distilled-water for 24 hr. The starved flies were temporarily anesthetized on ice for 15–30 s and transferred to a 35 mm diameter petri dish. There was a partition in the middle of the dish, and 1% agarose and 100 mM sucrose were added to both sides of the dish, with one side containing HMIs. Each side of the dish was labeled by either a blue dye (0.1 mg/mL Brilliant Blue FCF, Sigma) or a red dye (0.2 mg/mL Sulforhodamine B, Sigma). Flies ate in the dark for 90 min, and the preferences of flies was calculated by observing the colors in their abdomens. NR, NB and NP denoted the number of fruit flies with red, blue and purple abdomens respectively. A fly that ate both blue and red dyes had a purple abdomen. To quantitatively analyze the feeding preferences, we defined the Avoidance Index (AI) as AI = (NR - NB) / (NR + NB + NP) (ions in the side of blue dye) or AI = (NB - NR) / (NR + NB + NP) (ions in the side of red dye). An AI of 1 indicates that all flies avoid food containing metal ions, while an AI of 0 indicates that there is no preference for the two kinds of food. To eliminate the preference for dyes, metal ions were tested with both dyes.

#### Two-choice positional assay

Similar to the two-choice feeding test, 3–5-day-old flies were pre-starved for 24 hr, and quickly transferred to a 60 mm-diameter dish for testing after brief anesthesia by freezing. Both sides of the dish contained 1% agarose and 100 mM sucrose. Different concentrations of metal ions were added to one side of the dish. We recorded the location of flies for 2 hr with 15 sec/frame and analyzed the distribution of flies with a custom MATLAB script. The distribution at the end of 2 hr was used as the indicator of positional choice. Positional AI was defined as Positional AI = (NC - NE) / (NE + NC). NE or NC indicates the number of flies on the side without or with metal ions. The walls and lid of the dish were pretreated with Sigmacote to restrict the flies to stay on the agarose surface at the bottom.

#### Proboscis extension response (PER)

The PER assay was modified from a standard protocol.^68^ Flies that were starved for 24 hr were temporarily anesthetized on ice and fixed on a cover slide from the back with light-curable glue and recovered in a wet box for 2 hr. Before the test, flies were fed with distilled water to eliminate the influence of thirst. First, 100 mM sucrose was given to confirm that the animals were able to extend a proboscis. Flies that were not able to extend a proboscis were discarded. Next, filter paper containing sucrose solution and HMIs was given to the labellum or foreleg of flies. We touched the proboscis or foreleg with filter paper and then withdrew it quickly. Each solution was tested three times, and the labellum or foreleg was wiped with distilled water between each test. The proboscis extension probability of three tests was calculated to represent the perception of flies to the test solution.

#### Calcium image

Female flies were raised at 25 °C for 2–3 days. A fly was immobilized by inserting an electrode into the thorax and extending it towards the labellum,^69^ and then it was transferred to a fixed platform to observe the GRNs in the labellum using a confocal microscope.^42^ Milli-Q water or an aqueous solution containing metal ions was circulated via a pump at a speed of 3.8 mL/min. Firstly, the fixed flies were recorded in Milli-Q water for 6–8 min. The stimulus of metal ions or alkaloid were presented for 3–4 min and washed away with water until the fluorescence decreased to the basal level. GCaMP and tdTomato were expressed in target neurons simultaneously. Images were acquired as timelapse 3D (XYZ) stacks using a Leica SP8 confocal microscope with a 40× water-immersion objective (NA = 0.8), then were aligned and processed in MATLAB.

For analysis, the fluorescence intensities were calculated separately for individual neurons. First, the red (mtdTomato) and green (GCaMP) channels of raw data were z-projected (maximum intensity projection). The projected image from a stack was calculated as a frame. The ROI (region of interest) region enclosing each neuron was manually defined based on tdTomato expression. Fluorescence intensities of the red and green channels in each ROI were calculated, and the corresponding normalized fluorescence intensity was derived from F = F_green_/F_red_. The mean fluorescence intensity of 15 frames before the stimulus was designated as F0. Changes in the fluorescence intensity of each labeled neuron at time t were calculated as: ΔF/F0 = (F_t_ - F0)/F0. Sample traces revealed ΔF/F0 values over time from all labeled neurons in the labellum of a single fly, with each trace corresponding to a single neuron. Peak responses after stimulation in individual neurons were calculated for each stimulus. KCl solution (0.1 M) was added at the end of the experiment to assess the condition of the neurons. Different GCaMPs was used due to limited available transgenic sites for generating flies of specific genotypes. Within each set of experiments, the GCaMP6 variant was always the same in both the control and experimental groups to ensure a direct comparison of the results.

#### Immunohistochemistry and imaging

The heads of 5–7-day-old flies were removed and treated with a transparent solution containing 80% glycerol and 20% PBST (PBS with 0.5% Triton) for 1.5–2 hr and mounted with Vectashield solution (Vector Labs Inc.). The dissected brains removed labellum and legs of flies were fixed in 4% PFA (Paraformaldehyde) for 3–4 hr, then washed in PBST 3 times for 15 min each and mounted with a mounting solution. Images were acquired with a Leica SP8 with a 20× objective at a resolution of 1024 × 1024 and processed with ImageJ.

#### Food-intake assay

Food-intake assays were modified from the previous description.^66^ Briefly, twenty 5–7-day-old female flies were tested in a vial filled with 1% agarose, sucrose (100 mM), blue food dye (Erioglaucine Disodium Salt), and various HMIs for 48 hr and frozen at -80°C to stop feeding. Flies were transferred to 1.5 mL Eppendorf tubes containing 500 μL of degrading buffer (1% PBST) and centrifuged at 13,000 rpm for 30 min. The supernatant was placed in a 96-well plate and its absorbance at 630 nm was measured to calculate feeding amounts.

#### Survival assay

Survival assays were referred to the previous description.^39^ A total of 20 newly enclosed female flies were collected and fed in a medium containing 1% agarose, 100 mM sucrose, and 20 μM HMIs or alkaloids. The food was changed every 2 days and the number of surviving flies was counted every day.

### Quantification and statistical analysis

#### Data statistics and analysis

Data analysis and result plots in the experiments were mainly completed in MATLAB (2018a, MathWorks) and GraphPad Prime 8 (GraphPad Software, Inc). K-means clustering algorithm was used for data partition. All error bars represent the standard error of the means (SEM). Differences between groups were analyzed by Student’s t-test (two-sided) or one-way ANOVA with Tukey’s post hoc test. *P* > 0.05 was considered non-significant. *∗P* < 0.05, *∗∗P* < 0.01, and *∗∗∗P* < 0.001 were considered significant.

## Data Availability

All data have been deposited at Mendeley Data, and are publicly available as of the date of publication. DOI is listed in the key resources table. This paper does not generate original code. Any additional information required to reanalyze the data reported in this paper is available from the lead contact upon request.
