# Supplementary material for: Taste coding of heavy metal ion-induced avoidance in Drosophila
Source: iScience. 2023 Apr 7;26(5):106607. doi: 10.1016/j.isci.2023.106607 (PMC10148117; doi:10.1016/j.isci.2023.106607)
Supplement: Document S1. Figures S1–S6 [file mmc1.pdf]

iScience, Volume *26*

Supplemental information

Taste coding of heavy metal ion-induced avoidance in *Drosophila*

Xiaonan Li, Yuanjie Sun, Shan Gao, Yan Li, Li Liu, and Yan Zhu

Fig. S1. Concentration-dependent aversive response to heavy metal ions in two choice feeding assay, related to Figure 1.

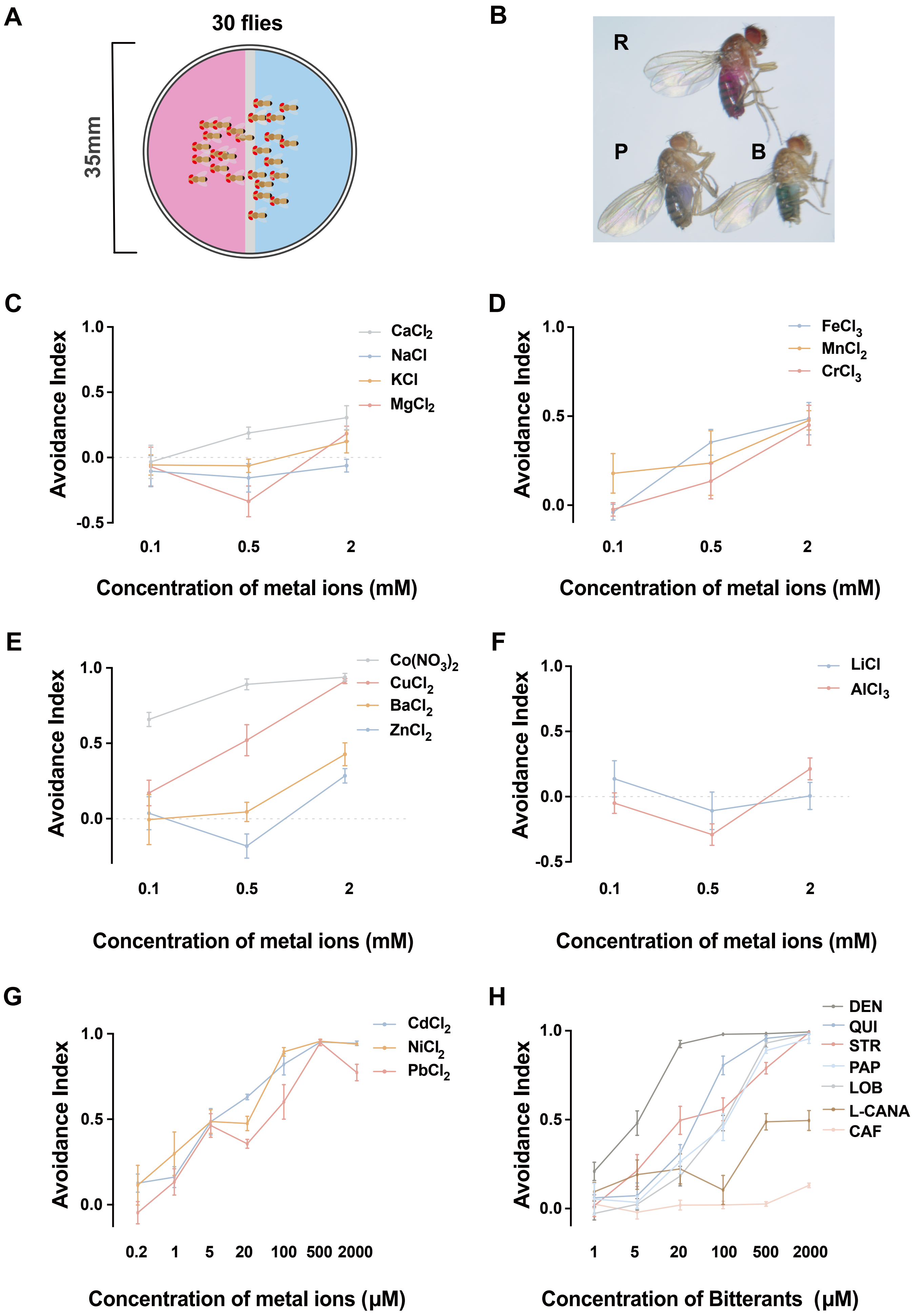

Figure S1. Concentration-dependent aversive response to heavy metal ions in two choice feeding assay, related to Figure 1.

(A) Schematics showing the dish for a two-choice feeding assay. The food on both sides contains a different color, while only the food on one side contains metal ions.

(B) Colored abdomens of flies at the end of two-choice feeding. R, B, and P represent flies with red, blue, and purple abdomens, respectively. Fruit flies with purple abdomens ate food containing both blue and red dyes.

(C-G) Quantification of *Canton-S* avoidance of food containing Na^{+}, K^{+}, Ca^{2+}, or Mg^{2+} (A, N = 4-8), Fe^{3+}, Mn^{2+} ,or Cr^{3+} (B, N = 6–10), Zn^{2+}, Ba^{2+}, Cu^{2+}, or Co^{2+} (C, N = 8-12), Li^{+}, or Al^{3+} (D, N = 8–10), and Cd^{2+}, Ni^{2+}, or Pb^{2+} (E, N = 8-12) of different concentrations.

(H) Quantification of *Canton-S* avoidance of food containing various bitterants of different concentrations. N = 8.

The lines and the error bars indicate mean +/- SEM.

Figure S2. Positional aversive response to heavy metals and bitter compounds are shown in flies, and repulsion are ubiquitous in *Drosophilidae*, related to Figure 1.

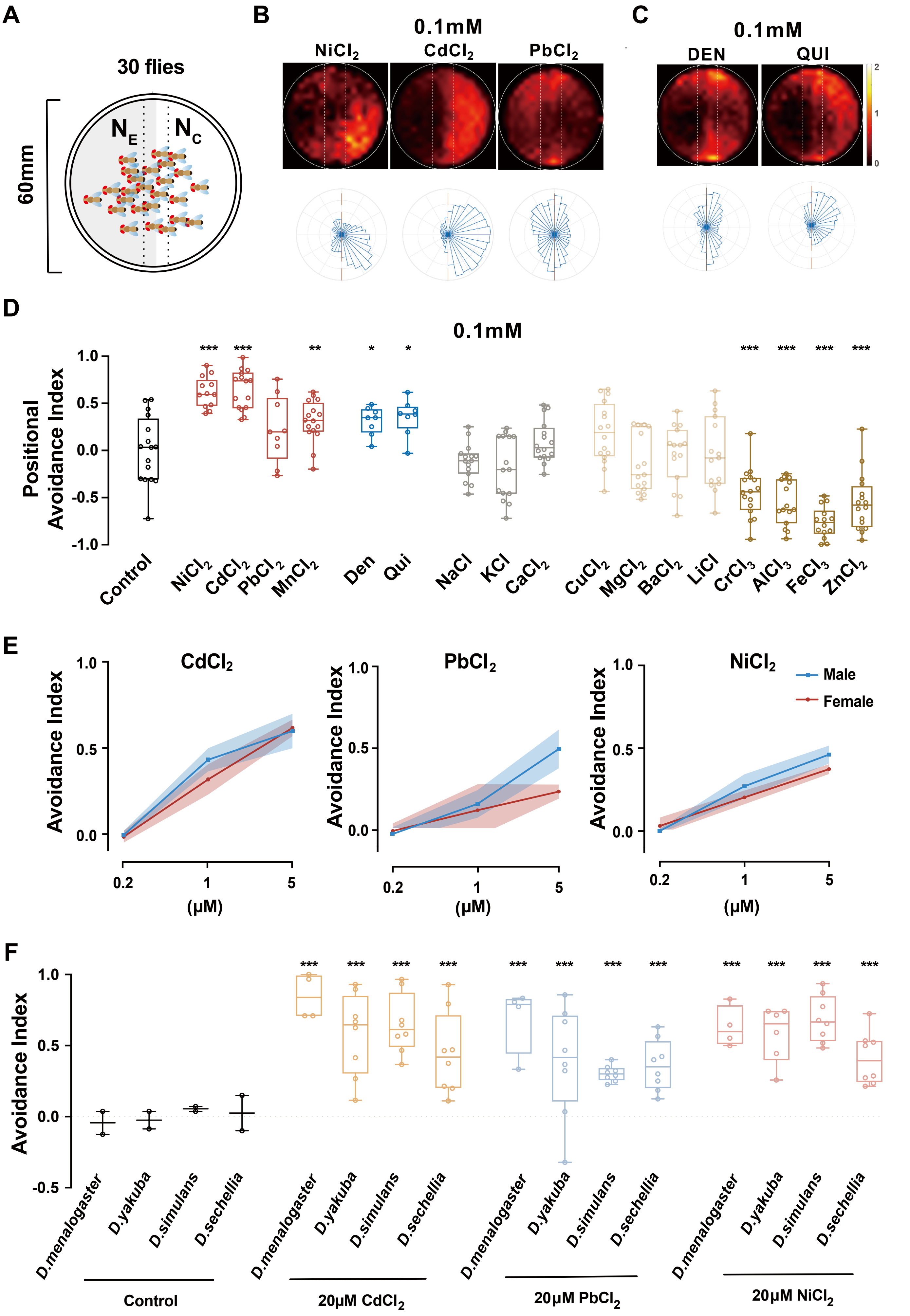

Figure S2. Positional aversive responses to heavy metals and bitterants are shown in wild-type flies, and repulsive responses are ubiquitous in *Drosophilidae*, related to Figure 1.

(A) Schematics showing the behavioral paradigm for positional avoidance. Two imaginary lines divide the dish into three areas. N_C indicates the number of flies on the blank side (without heavy metals, right), N_E indicates the number of flies on the side containing heavy metal ions (left). Flies that stayed in the middle between the two lines were not taken into account for analysis.

(B–C) Pseudo-color heat maps (top) and polar plots (bottom) presented the distributions of flies in the dishes with heavy metal ions (B) or bitterants (C).

(D) Quantification of positional preference to various metal ions and bitterants. Metal ions were colored as follows: essential metals (grey), trace metals (brown, aversive trace metals were colored by dark brown), toxic heavy metal ions and heavy metals eliciting strong aversions (red), and bitterants (blue). N = 8–16.

(E) Quantifying the difference between male (blue) and female (red) *Canton-S* flies in the feeding avoidance of Cd^{2+} (A), Pb^{2+} (B), Ni^{2+} (C). The bold lines and shaded areas indicate mean +/- SEM. N = 8.

(F) Evaluating different species for their feeding avoidance of heavy metals. N = 4–8.

In the control group, the food had no added metal ions. Box and whisker plots in D and F: the scatter points show all data points; the box includes the 25th to 75th percentile, and the line in the box shows the median of the data set. One-way ANOVA followed by Tukey's post hoc test for multiple comparisons. $^*P < 0.05$, $^{**}P < 0.01$, $^{***}P < 0.001$.

Figure S3. The gustatory system mediates the sensation of heavy metals, related to Figure 2.

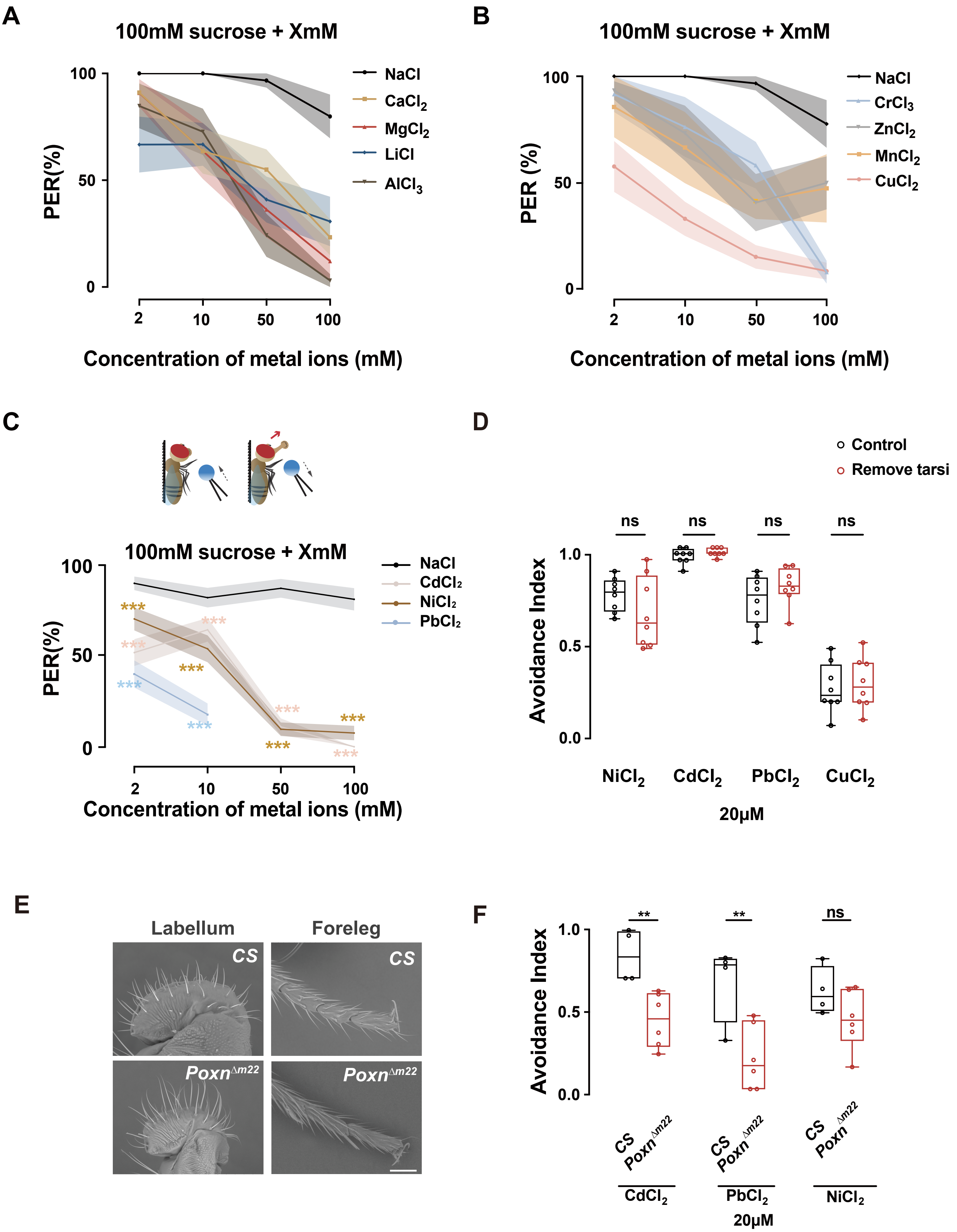

Figure S3. The gustatory system mediates the sensation of heavy metals, related to Figure 2.

(A) Effects of the metal ions (Na^{+}, Li^{+}, Ca^{2+}, Mg^{2+}, and Al^{3+}) with indicated concentrations on the proboscis extension response (PER) in starved flies when applied to the labellum. The bold line and shaded region indicate mean +/- SEM. N = 10–16.

(B) Effects of the heavy metal ions (Cu^{2+}, Zn^{2+}, Mn^{2+}, and Cr^{3+}) with indicated concentrations on PER in starved flies when applied to the labellum. The bold line and shaded region indicate mean +/- SEM. N = 6–12.

(C) Behavioral effects elicited by application of heavy metal ions to tarsi. Top: schematic of proboscis extension reflex (PER) with stimulation on foreleg tarsi. The tip of filter paper containing 100mM sucrose with various metal ions briefly touched the foreleg then withdrew, and the extension of the proboscis was monitored. Bottom: quantifying the rate of PER frequency of starved flies to sucrose solution containing different concentrations of various metal ions. The bold line and shaded region indicate mean +/- SEM. N = 8–10.

(D) Comparing the feeding preferences to food containing heavy metal ions between intact flies and flies with tarsi of all legs removed. N = 8.

(E) Scanning electron micrographs showing the morphology of proboscis and legs of wild-type (*Canton-S*) and $Poxn^{\Delta m22}$ flies. The mechanosensory hairs transformed from chemosensory hairs in $Poxn^{\Delta m22}$ flies appeared longer and thinner than the normal chemosensory hairs in wild-type flies. Scale bar: 50 μm.

(F) Comparing the preferences of feeding between wild-type (*Canton-S*) and $Poxn^{\Delta m22}$ flies to food containing 20 μM heavy metal ions. N = 4–6.

Statistical analysis made comparisons between two groups at the same concentration in (C), compared between mutants and control groups in (F). Student's t-test for comparisons between two groups. ns: $P > 0.05$, $^{**}P < 0.01$, $^{***}P < 0.001$.

Figure S4. Validation of imaging paradiam via response of *Gr66a*⁺ neuorns to DEN, and quantifying the physiological performances of flies pre-exposed to Cd^{2+}, related to Figures 2-3.

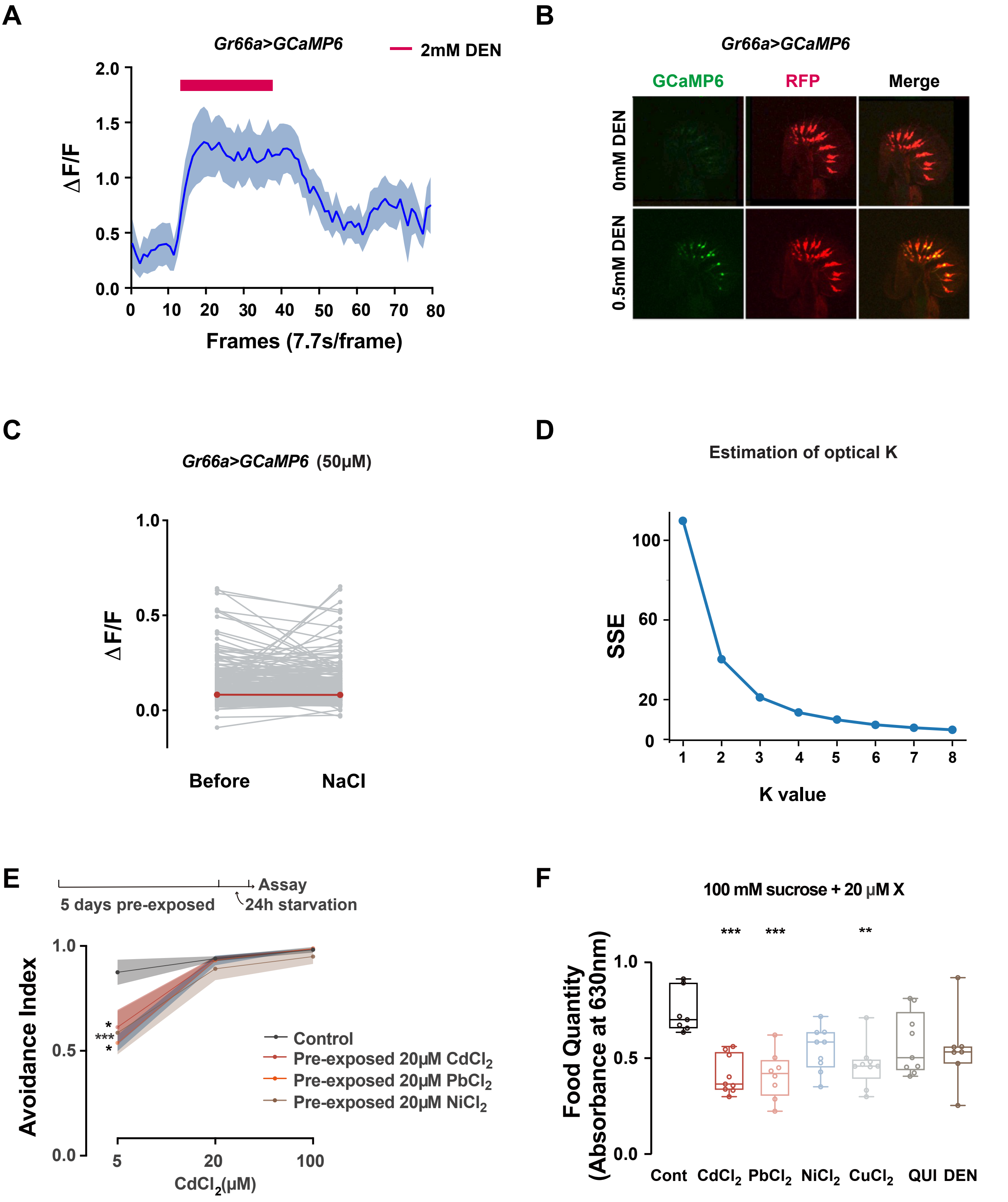

Figure S4. Validation of imaging paradigm via response of *Gr66a*$^+$ neurons to DEN, and quantifying the physiological performances of flies pre-exposed to Cd^{2+}, related to Figures 2-3.

(A) Representative trace of Ca^{2+} response of Gr66a$^+$ neurons to 2 mM bitterant Denatonium. The bold line and shaded region indicate mean +/- SEM. Red bar indicates the application of Denatonium.

(B) Representative images of Ca^{2+} response (Green) of *Gr66a*$^+$ neurons to 0.5 mM Denatonium. Genotype: *Gr66a-Gal4/+; UAS-GCaMP6f, UAS-mtdTomato*.

(C) Fluorescence intensity of GCaMP signals in *Gr66a*$^+$ neurons before and after NaCl (50 μM) application. N = 11. The red trace represented mean value.

(D) Comparing the sum of squared error (SSE) for different K values in K-means cluster analysis prior to Figure 2C. From this, a K value of 3 was chosen in the location where the graph decreased abruptly (the elbow).

(E) Quantifying Cd^{2+} avoidance after flies were pre-exposed to heavy metal ions (20 μM) for 5 days. Top: a diagram showing the experimental timeline. Bottom: after pre-exposure, flies were evaluated for their avoidance of Cd^{2+} at 5, 20, or 100 μM. N = 4. The lines and shaded regions indicate mean +/- SEM.

(F) Quantifying the amount of food in the digestive tract after flies were maintained on the media containing heavy metal ions or bitter agents for 48 hours. N = 6–8.

Statistical analyses compared stimulus and control groups at the same concentration in (E),. In the control group of (F), the food had no added metal ions. One-way ANOVA followed by Tukey's post hoc test for multiple comparisons in (F) and significant differences are marked: $*P < 0.05$, $**P < 0.01$, $***P < 0.001$.

Figure S5. Mutants of gustatory receptors and TRP channels all exhibit normal avoidance of heavy metal ions, whereas ionotropic receptors (IR76b, IR25a, and IR7a) are non-selective mediators of heavy metals, related to Figure 4.

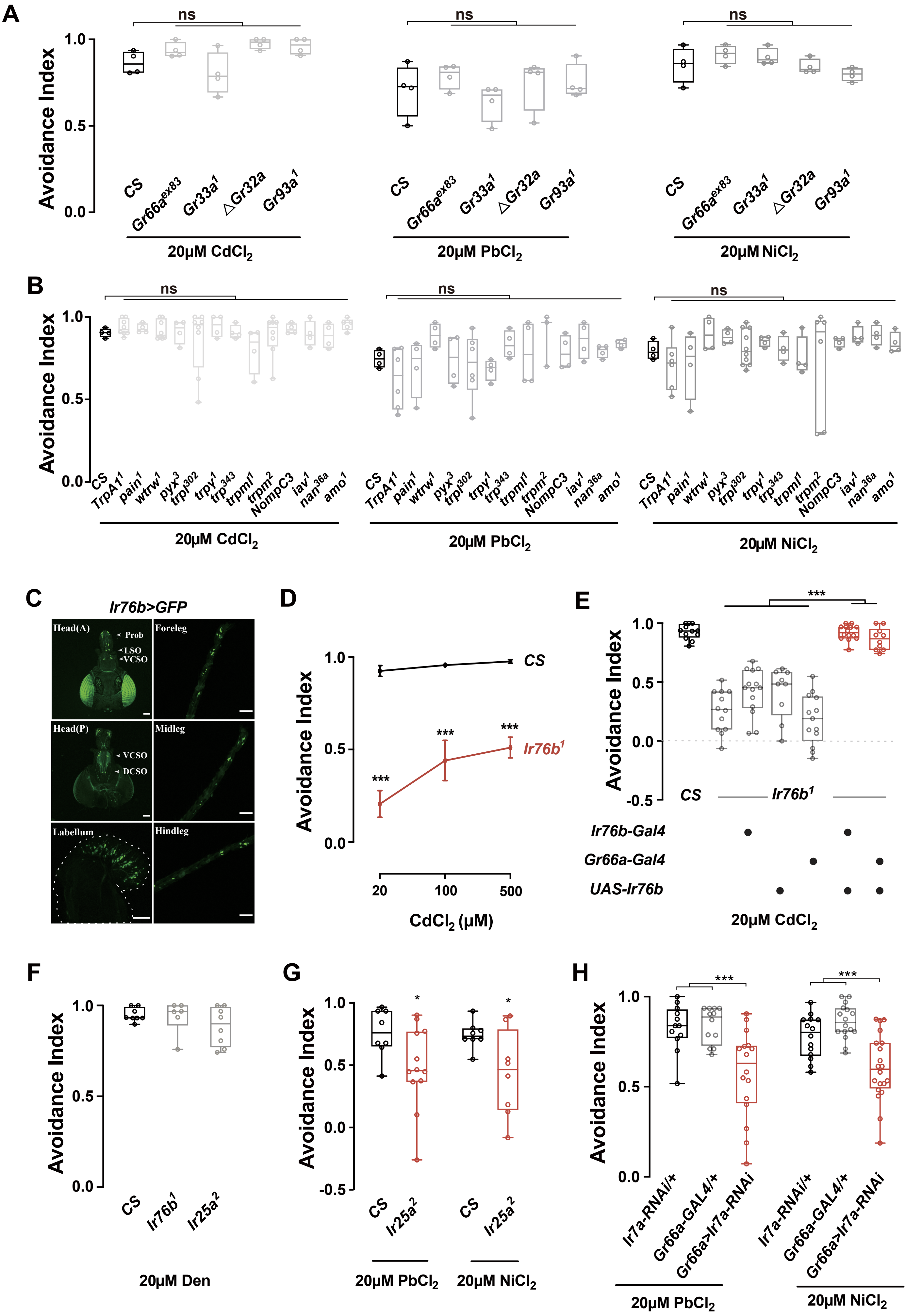

Figure S5. Mutants of gustatory receptors and TRP channels all exhibit normal avoidance of heavy metal ions, whereas ionotropic receptors (IR76b, IR25a, and IR7a) are non-selective mediators of heavy metals, related to Figure 4.

(A) Gustatory receptor mutant avoidance of 20 μM heavy metal ions. *Canton-S* (*CS*) was the wild-type control. N = 4.

(B) TRP channel mutant avoidance of 20 μM heavy metal ions. *Canton-S* (*CS*) was the wild-type control. N = 4–8.

(C) Expression of *Ir76b*$^{+}$ GRNs in labellar, tarsal, and pharyngeal taste organs. Arrows refer to different groups of GRNs in the head. P: posterior view of the head, A: anterior view of the head, Prob: Proboscis. The labral sense organ (LSO), ventral cibarial sense organ (VCSO), and dorsal cibarial sense organ (DCSO) present three distinct internal taste organs in the pharynx. Scale bar: 50 μm. Strong autofluorescence was observed in the eyes, head, and joints of the legs.

(D) Avoidance responses of *Ir76b*^{1} mutants to different concentrations of Cd^{2+}. Data are represented as mean +/- SEM. N = 8.

(E) Behavioral responses of *Ir76b*^{1} flies with *Ir76b* overexpressed in *Gr66a*$^{+}$ or *Ir76b*$^{+}$ neurons to 20 μM Cd^{2+} in a food-choice test. N = 10–14.

(F) Quantification of the avoidance responses of *Ir76b* or *Ir25a* mutants to 20 μM denatonium in the food preference assay. N = 6–8.

(F) Behavioral responses of *Ir25a* mutants to food containing 20 μM Pb^{2+} or Ni^{2+}. N = 8–12.

(G) Behavioral responses of flies with *Ir7a* knocked down specifically in the *Gr66a*$^{+}$ neurons via RNA interference to 20 μM Pb^{2+} or Ni^{2+}. N = 12–20.

Student's t-test for comparisons between each mutant and wild-type control (*CS*) in (F) and (G), also between two groups at the same concentration in (D). One-way ANOVA followed by Tukey's post hoc test for multiple comparisons in (A), (B), (E) and (H). ns: $P > 0.05$, $*P < 0.05$, $**P < 0.01$. $***P < 0.001$.

Figure S6. *Ir47a*[+] neurons, which largely co-localized with *ppk23*[+] neurons, are necessary for Cd^{2+} repulsion, related to Figures 5-6.

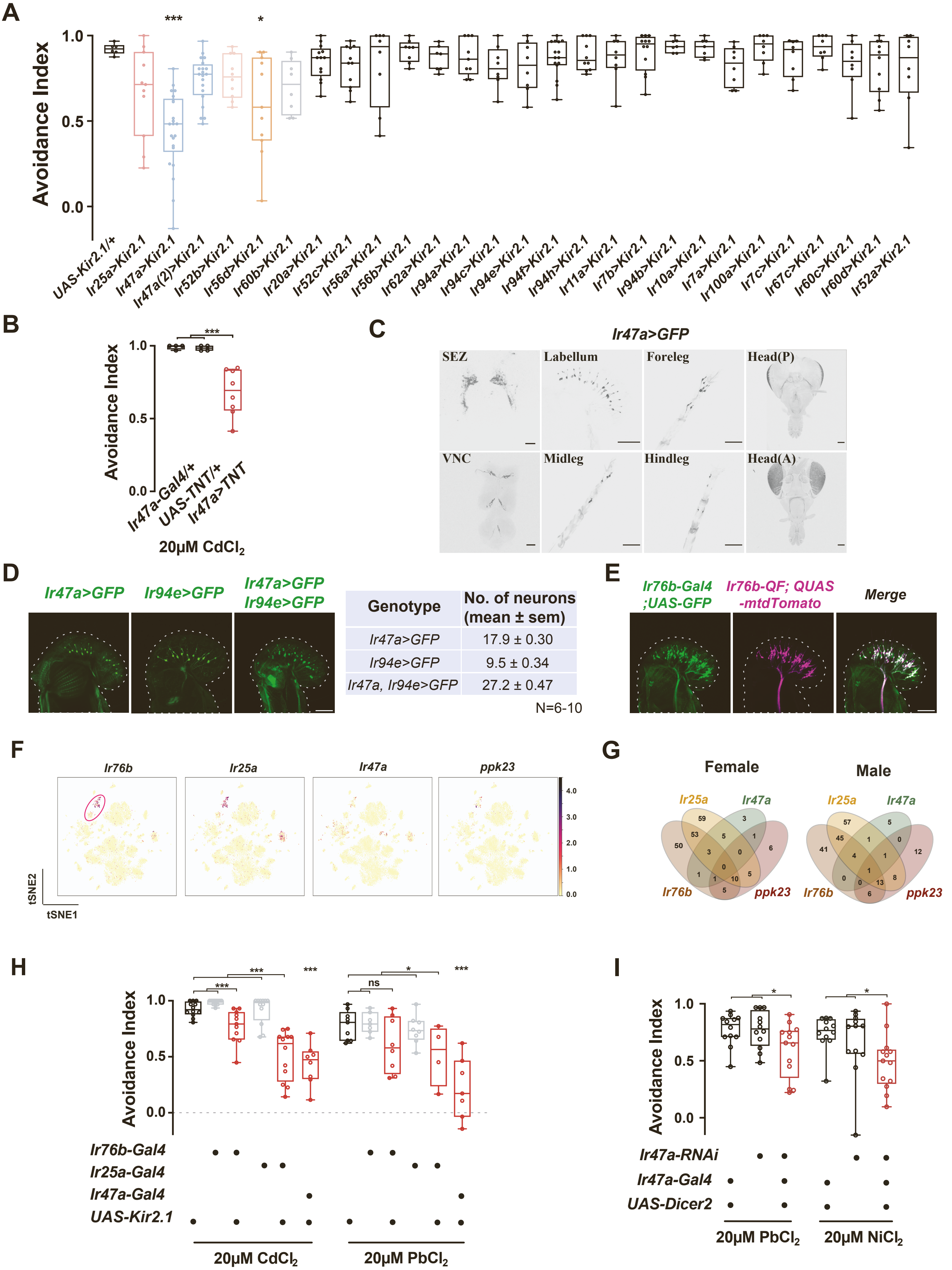

Figure S6. *Ir47a*$^+$ neurons, which largely co-localized with *ppk23*$^+$ neurons, are necessary for Cd^{2+} repulsion, related to Figures 5-6.

(A) A behavioral screen of Gal4 lines for ionotropic receptor-expressing neurons for their involvement in Cd^{2+} avoidance, using *UAS-Kir2.1* as the effector. N = 6–22.

(B) Behavior response to 20 μM Cd^{2+} in flies with *Ir47a-Gal4* labeled neurons silenced via *UAS-TNT*. N = 4–8.

(C) Expression patterns of *Ir47a*$^+$ neurons in the brain, ventral nerve cord (VNC), labellum, tarsi, and head (anterior (A) and posterior view (P)). Darker areas indicate stronger expression signals. Scale bars in brain, VNC, labellum and legs: 50 μm. Scale bars in Head (P) and Head (A): 100 μm.

(D) Statistical analysis of the number of labellar neurons labelled by *Ir47a-Gal4* or *Ir94e-Gal4*. Top: Expression patterns of *Ir47a-Gal4*, *Ir94e-Gal4* and their combination. Genotypes from left to right: *Ir47a-Gal4/+; UAS-GFP/+* (left), *UAS-GFP/+; Ir94e-Gal4/+* (middle), and *Ir47a-Gal4/ UAS-GFP; Ir94e-Gal4/UAS-GFP* (right). Right: summarization of the enumeration of neurons in each genotype.

(E) Co-expression of *Ir76b-Gal4* labeled neurons (green) and *Ir76b-QF* labeled neurons (magenta) in the labellum. Genotypes: *Ir76b-QF /UAS-GFP; Ir76b-Gal4/QUAS-tdTomato*. Scale bar: 50 μm.

(F-G) Visualization of the neurons expressing *Ir76b*, *Ir25a*, *Ir47a* and *ppk23* using scRNA-seq data derived from *Drosophila* proboscis and maxillary palp.

(F) Individual tSNE plots showing the neurons with various expression levels of *Ir76b*, *Ir25a*, *Ir47a*, and *ppk23*, respectively. There are a total of 26301 neurons with 12802 genes detected in this data set. The color bar indicated the expression levels (0-4) of each indicated gene. The labellar neurons cluster is bounded by a red circle in the Ir76b panel, and the labellar neurons with expression levels higher than or equal to 1.0 were used for further analysis in (F).

(G) Venn diagram displaying the overlap of neurons with different expression profiles relevant to the *Ir76b*, *Ir25a*, *Ir47a* and *ppk23* genes in male or female flies. Neurons with *Irs/ppk23* expression greater than 0.0 were considered as *Irs/ppk23* positive neurons as assessed with results in (E).

(H) Evaluating behavioral responses to 20 μM of Cd^{2+} or Pb^{2+} in flies with the indicated labelled neurons suppressed by Kir2.1. N = 4–12.

(I) Behavioral responses to 20 μM Pb^{2+} or Ni^{2+} in flies with *Ir47a* knockdown specifically in *Ir47a*$^+$ neurons via RNA interference. N = 12–13.

Statistical analysis: One-way ANOVA followed by Tukey's post hoc test for multiple comparisons in (A), (B), (H) and (I). Significant differences are marked: $*P < 0.05$, $**P < 0.01$, $***P < 0.001$.
